# FSS-ULivR: a clinically-inspired few-shot segmentation framework for liver imaging using unified representations and attention mechanisms

**DOI:** 10.1007/s00432-025-06256-0

**Published:** 2025-07-17

**Authors:** Ripon Kumar Debnath, Md. Abdur Rahman, Sami Azam, Yan Zhang, Mirjam Jonkman

**Affiliations:** 1 Department of Computer Science and Engineering, United International University, Dhaka, 1212 Bangladesh; 2 Faculty of Science and Technology, Charles Darwin University, Northern Territory 0909 Darwin, Australia

**Keywords:** Few-shot learning, Medical image segmentation, Prototype-based learning, Transformer networks, Cross-dataset evaluation

## Abstract

Precise liver segmentation is critical for accurate diagnosis and effective treatment planning, serving as a foundation for medical image analysis. However, existing methods struggle with limited labeled data, poor generalizability, and insufficient integration of anatomical and clinical features. To address these limitations, we propose a novel Few-Shot Segmentation model with Unified Liver Representation (FSS-ULivR), which employs a ResNet-based encoder enhanced with Squeeze-and-Excitation modules to improve feature learning, an enhanced prototype module that utilizes a transformer block and channel attention for dynamic feature refinement, and a decoder with improved attention gates and residual refinement strategies to recover spatial details from encoder skip connections. Through extensive experiments, our FSS-ULivR model achieved an outstanding Dice coefficient of 98.94%, Intersection over Union (IoU) of 97.44% and a specificity of 93.78% on the Liver Tumor Segmentation Challenge dataset. Cross-dataset evaluations further demonstrated its generalizability, with Dice scores of 95.43%, 92.98%, 90.72%, and 94.05% on 3DIRCADB01, Colorectal Liver Metastases, Computed Tomography Organs (CT-ORG), and Medical Segmentation Decathlon Task 3: Liver datasets, respectively. In multi-organ segmentation on CT-ORG, it delivered Dice scores ranging from 85.93% to 94.26% across bladder, bones, kidneys, and lungs. For brain tumor segmentation on BraTS 2019 and 2020 datasets, average Dice scores were 90.64% and 89.36% across whole tumor, tumor core, and enhancing tumor regions. These results emphasize the clinical importance of our model by demonstrating its ability to deliver precise and reliable segmentation through artificial intelligence techniques and engineering solutions, even in scenarios with scarce annotated data.

## Introduction

Liver cancer has become the sixth most frequently diagnosed cancer and remains the third leading cause of cancer-related death worldwide, with 865,269 new cases and 757,948 deaths in 2022 (Bray et al. 2024). Precise segmentation helps clinicians identify and monitor liver cancers, allowing more timely and effective treatments. However, achieving high-quality segmentation can be challenging due to variation in tumor appearance and liver structures between patients and imaging modalities (Alksas et al. 2021).

Recent advances in neural network architectures have significantly improved the precision of liver and tumor segmentation. Hybrid networks such as Residual U-Net (ResUNet) (Rahman et al. 2022) and Hybrid Densely Connected UNet (H-DenseUNet) (Li et al. 2018) use encoder-decoder structures with residual and dense connections to enhance the extraction of features. These models combine local and global information to better capture liver tumor structures. Generative models such as partial convolution generative adversarial network (PCGAN) (Liu et al. 2023) help generate synthetic liver lesions, improving the robustness of the model. Attention-based methods (Hettihewa et al. 2023; Seo et al. 2019; Jiang et al. 2019), like Attention Hybrid Connection Net (AHCNet), use attention with skip connections to increase segmentation performance. Deformable encoder-decoder network (DefED-Net) (Lei et al. 2021) applies deformable convolutions with a spatial pyramid module for better context learning. Three-dimensional dual path multiscale convolutional neural network (TDP-CNN) (Meng et al. 2020) balances performance and efficiency using multiscale and deformable convolutions. Semi-supervised approaches (Chen et al. 2024; Alsaleh et al. 2024), such as Adapting SAM in the loop for semi-supervised liver tumor segmentation (ASLseg) and Model-Agnostic Meta-Learning (MAML), reduce the dependency on large-labeled datasets by using adapted or short-shot supervision. Multiscale methods (Zhang et al. 2022; Gao et al. 2023), such as Decoupled pyramid correlation network (DPC-Net) and Laplacian Salience-Gated Feature Pyramid Network improve accuracy by integrating features at different scales with attention and salience filters.

Despite their progress, many state-of-the-art liver segmentation approaches still face several limitations. Although hybrid networks and deformable convolutions improve feature learning, they often fail to capture the complex anatomical variations of liver structures. Generative models help address data scarcity but do not always produce clinically reliable variations. Attention mechanisms, salience filters, and spatial pyramid modules improve focus and context but are often insufficient to highlight the most relevant regions for segmentation tasks. Semi-supervised and few-shot methods reduce the need for large datasets, yet they still struggle to maintain robust performance when labeled data is extremely limited. Furthermore, many models have high computational costs, making them unsuitable for real-time or resource-constrained environments, and they often perform poorly on unseen datasets, limiting their clinical applicability without additional fine-tuning.

**Fig. 1:**
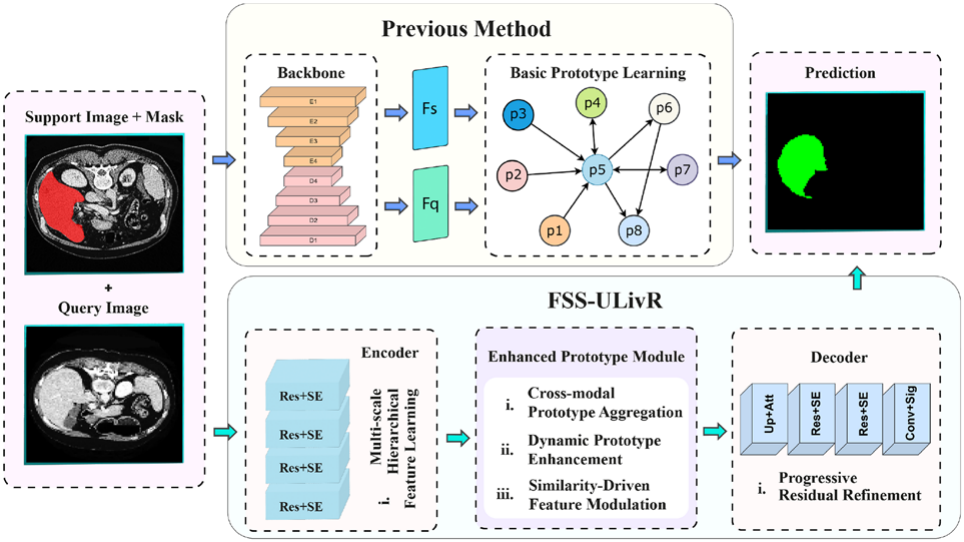
The overview of our FSS-ULivR framework compared to previous approaches. The previous method employs basic prototype learning with backbone feature extraction and simple prototype relations. Our proposed FSS-ULivR enhances the few-shot segmentation pipeline with an advanced encoder using multi-scale hierarchical feature learning, an Enhanced Prototype Module featuring cross-modal prototype aggregation, dynamic prototype enhancement, and similarity-driven feature modulation, followed by a decoder with progressive residual refinement

To address these challenges, we introduce Few-Shot Segmentation with Unified Liver Representation (FSS-ULivR), a model that utilizes a unified liver representation to learn robust features across support and query images, enabling accurate segmentation with minimal annotations, as illustrated in Fig. 1. Our approach incorporates a ResNet encoder integrated with Squeeze-and-Excitation (SE) blocks to effectively capture both global and fine-grained features. A prototype-based module further refines the support-query relationships using attention mechanisms, while the decoder employs improved attention gates and residual refinement to better recover spatial details and produce more accurate segmentation boundaries. Our approach demonstrates strong generalization across datasets, with lower memory usage, making it suitable for real-world clinical environments. The major contributions of our framework are as follows.

- FSS-ULivR is a few-shot segmentation framework specifically designed for liver segmentation, employing a 1-shot episodic learning strategy in which each episode comprises a single annotated support image–mask pair and an unlabeled query image, utilizing this limited supervision to accurately predict the corresponding query mask.
- The encoder utilizes residual blocks augmented with SE modules to recalibrate channel features and preserve spatial hierarchies, thereby enhancing feature learning from both support and query images for improved segmentation performance.
- An Enhanced Prototype Module is introduced to compute support prototypes from masked data and to fuse refined support and query features using transformer self-attention and channel-attention mechanisms, thereby boosting few-shot segmentation accuracy.
- The decoder incorporates enhanced attention gates, residual refinement, and multiscale skip connections to restore spatial detail and generate accurate liver segmentation boundaries.
- Extensive cross-dataset evaluations and ablation studies demonstrate the generalizability and robustness of FSS-ULivR. The model is trained on the LiTS dataset and evaluated on 3DIRCADB01, CRLM, CT-ORG, and MSD-Task03-Liver, achieving high Dice coefficients, IoU, and specificity for liver segmentation while maintaining computational efficiency. Additional evaluations on CT-ORG for multi-organ segmentation and BraTS 2019 and 2020 for brain tumor segmentation demonstrate consistently superior performance across diverse anatomical structures and imaging modalities.

This article is organized as follows: In Sect. 2, we explore previous research on segmentation methods, focusing on the difficulties encountered in few-shot learning. Section 3 presents the FSS-ULivR model, highlighting its architectural components and advantages. Section 4 details the experimental setup, including datasets, preprocessing, and evaluation criteria. In Sect. 5, we evaluate the segmentation capabilities of the model, including its generalization to unseen datasets. Section 6 discusses the implications of our findings and their relevance to current research, including potential future directions. Finally, Section 7 summarizes our findings and concludes the study.

## Related work

### Hybrid convolutional neural networks

Recent advances in liver segmentation have been driven by hybrid architectures that integrate different deep-learning models to improve feature representation and segmentation accuracy. These models often combine elements from U-Net-based architectures (Rahman et al. 2022; Tran et al. 2020; Tan et al. 2021) and densely connected networks (Li et al. 2018) together with other convolutional enhancements (Balasubramanian et al. 2023; Lei et al. 2021; Meng et al. 2020; Ahmad et al. 2022; Hussain et al. 2025; Alam et al. 2024) to improve spatial and contextual understanding. Several studies have explored hybrid architectures to improve liver segmentation.

For example, Rahman et al. (2022) introduced a hybrid ResUNet architecture that integrates ResNet with U-Net, offering an effective solution for segmentation tasks. The model achieved a Dice Similarity Coefficient (DSC) of 99.2% on abdominal CT scans. On the other hand, Tran et al. (2020) extended U-Net into Un-Net using an n-fold convolutional unit as skip connections to enhance feature reuse. Their U2-Net and U3-Net obtained DSC scores of 96.38% and 73.69% for segmenting the liver and tumor in the LiTS dataset, respectively. Similarly, Li et al. (2018) proposed H-DenseUNet, which merges 2D and 3D DenseUNet architectures to incorporate both intra-slice and volumetric features. They achieved DSC scores of 98.2% and 93.7% for the liver and tumor segmentation tasks, respectively.

Further improvements have incorporated additional constraints and learning mechanisms. For example, in a study, Tan et al. (2021) combined a liver-shaped autoencoder with a segmentation network. Introducing hybrid loss functions for improvement, they achieved a DSC score of 82.55% on the Silver07 challenge. Furthermore, Lei et al. (2021) developed DefED-Net, which integrates deformable convolutions and multiscale spatial structuring, achieving Dice scores of 96.30% and 87.52% for the liver and tumors, respectively, on the LiTS dataset. Meanwhile, Balasubramanian et al. (2023) improved mask region-based convolutional neural networks (Mask R-CNN) with an adversarial propagation-based Swin Transformer network (APESTNet). APESTNet obtained Dice scores of 95.7% on LiTS and 97.31% on Sliver07. Similarly, Meng et al. (2020) introduced TDP-CNN that balances segmentation refinement with conditional random fields and achieved a Dice score of 94.6%. Also (Hussain et al. 2025) developed EFFResNet-ViT, a hybrid model combining EfficientNet-B0, ResNet-50, and a Vision Transformer (ViT) to fuse local and global features. It achieved 99.31% accuracy on BT CE-MRI and 92.54% on a retinal image dataset, with Grad-CAM and t-SNE enhancing interpretability. Furthermore, Alam et al. (2024) integrated YOLOv8 for ROI detection with ResNet50, SeResNet50, and ViT-B-16 for elbow fracture prediction. ViT-B-16 achieved 99% accuracy, demonstrating strong diagnostic potential in X-ray analysis. Ahmad et al. (2022) proposed a lightweight convolutional neural network with Gaussian weight initialization that uses three convolutional layers and two fully connected layers with softmax for classification to efficiently segment the liver from CT images.

However, hybrid convolutional models face key challenges. For instance, integration of multidimensional features can be problematic, as combining 2D and 3D data often leads to feature misalignment, with 2D features failing to capture 3D context and 3D features lacking fine-grained details. Additionally, preserving spatial consistency across slices remains a challenge, as hybrid models can struggle with maintaining smooth transitions between adjacent slices.

### Attention-based multi-scale feature fusion

To further improve segmentation accuracy, attention mechanisms and multi-scale feature integration have been incorporated into deep learning architectures. These methods refine feature selection by utilizing attention mechanisms (Jiang et al. 2019; Hettihewa et al. 2023; Seo et al. 2019; Kim et al. 2021; Hussain et al. 2025) and multi-scale integration strategies (Zhang et al. 2022; Ansari et al. 2022; Liu et al. 2024; Hussain and Shouno 2024) to capture fine-grained spatial details while suppressing irrelevant information.

For instance, Jiang et al. (2019) proposed AHCNet, which integrates soft and hard attention mechanisms along with skip connections, achieving a Dice score of 95.9% for the liver segmentation task. Similarly, Hettihewa et al. (2023) developed the Multi Attention Network (MANet), merging both channel and spatial attention features within a U-Net architecture, achieving a Dice score of 81.45%. Seo et al. (2019) introduced a modified U-Net (mU-Net) that utilizes object-dependent upsampling and improved residual skip connections, achieving a Dice score of 98.51% in the liver segmentation task. On the other hand, Kim et al. (2021) introduced a 3D few-shot segmentation model (FSSBiGRU) with a bidirectional GRU to enforce consistency across adjacent CT slices, coupled with transfer learning for target organ adaptation. Their approach achieved a Dice score of 88.7% for liver segmentation on the BCV dataset. Hussain et al. (2025) introduced DCSSGA-UNet, which utilizes DenseNet201 with channel spatial attention (CSA) and semantic guidance attention (SGA) to bridge semantic gaps. It achieved mDice scores of 98.85%, 95.71%, and 96.10% on CVC-ClinicDB, CVC-ColonDB, and Kvasir-SEG datasets, respectively. Likewise, Zhang et al. (2022) proposed DPC-Net, which utilized both spatial and semantic attention mechanisms for multi-level feature fusion, achieving a Dice score of 96.0% for liver tumor segmentation. Similarly, Ansari et al. (2022) proposed a fixed-width residual UNet backbone and Pyramid Atrous Convolutions networks for multiscale feature enhancement. Liu et al. (2024) presented a hierarchical inter-scale multi-scale feature fusion network for segmenting liver vessels with hierarchical inter-scale learning, which achieved a Dice score of 75.36% on the 3DIRCADb dataset. Additionally, Hussain and Shouno (2024) proposed MAGRes-UNet, incorporating multi-attention gate (MAG) modules and residual blocks within a U-Net. Using Mish and AdamW, it achieved 97.75% Dice on BT CE-MRI and 97.36% on HAM10000 skin lesion dataset.

These approaches often require extensive fine-tuning to adapt to different anatomical structures, and the computational demands of multi-scale and attention-based mechanisms hamper real-time deployment in clinical settings as well. Furthermore, the models’ performance often degrades when applied to data outside of the specific training sets.

### Generative and semi-supervised approaches

**Fig. 2:**
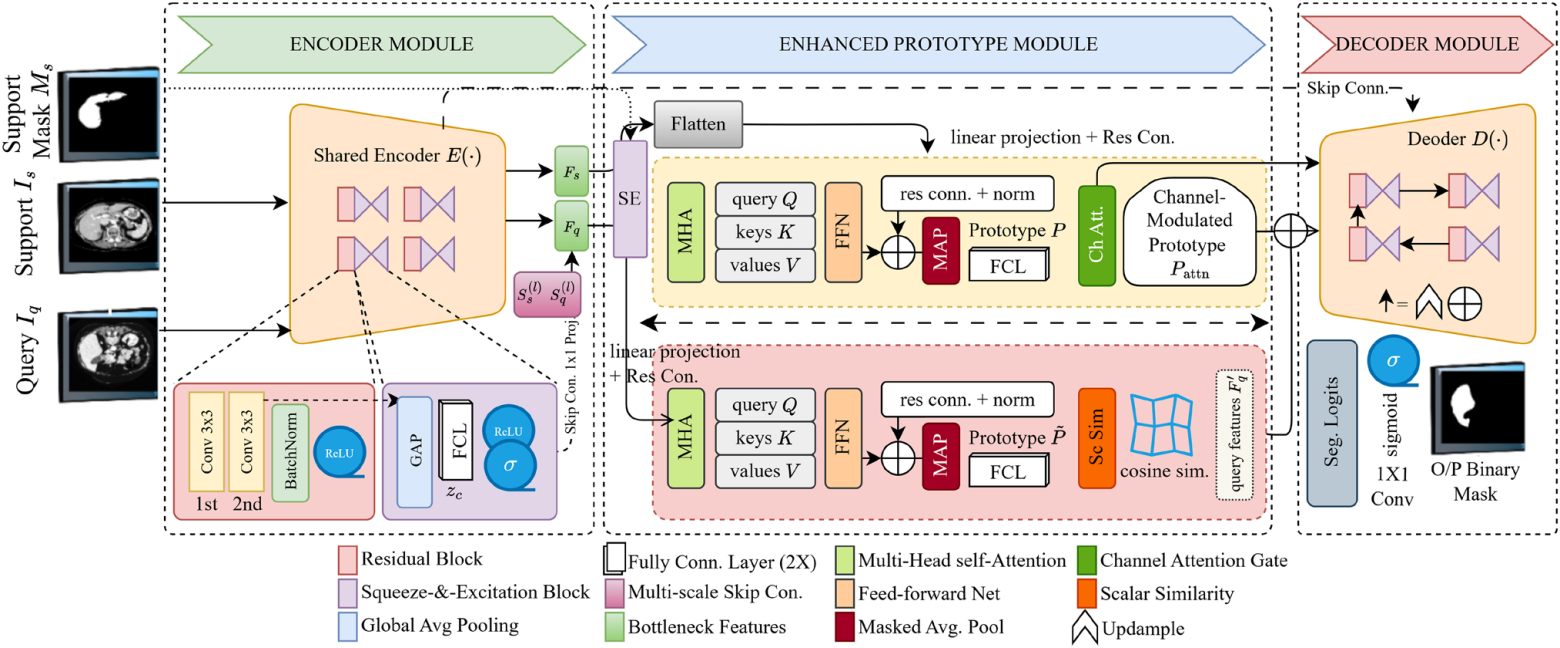
Illustration of the proposed FSS-ULivR Model, a few-shot segmentation framework that integrates a ResNet-based encoder, an enhanced prototype module with transformer and SE block, and a U-Net-based decoder with improved attention gate

Generative models and semi-supervised learning strategies have been used as effective solutions in scenarios where labeled medical data is scarce. These methods utilize synthetic data generation (Liu et al. 2023), adversarial learning (Zheng et al. 2022; Cheema et al. 2019; Awudong et al. 2024), and meta-learning (Alsaleh et al. 2024) along with semi-supervised techniques (Chen et al. 2024; Han et al. 2022; Huang et al. 2024) to improve segmentation performance with limited annotations.

For instance, Liu et al. (2023) developed PCGAN to generate synthetic liver lesions by integrating partial convolutions in a U-Net-like generator and employing a Wasserstein GAN with gradient penalty and spectral normalization for the discriminator. Similarly, Zheng et al. (2022) introduced a 4D deep learning model for segmenting hepatocellular carcinoma lesions, which integrates 3D convolution with convolutional long short-term memory. Cheema et al. (2019), on the other hand, further expanded on this idea with a Liver Extraction by employing Residual Convolutional Neural Networks. Their method achieved a DSC score of up to 92.10±3.4% on the Silver07 dataset.

In addition to generative approaches, semi-supervised learning has also made significant contributions to liver segmentation. For example, in a study, Chen et al. (2024) introduced the ASLseg framework, which adapts the SAM model to a semi-supervised setting by integrating domain-specific knowledge and utilizing pseudo-labels generated by a segmentation model. This adaptation achieved a Dice score of 74.28% on the LiTS dataset. Similarly. Huang et al. (2024) proposed a semi-supervised architecture with adaptive mask refinement that utilized a combination of labeled and large-scale unlabeled data and obtained a Dice score exceeding 94%. In another study, Awudong et al. (2024) proposed a prototype-based generative adversarial network (PG-Net) for few-shot liver segmentation, where a prototype-guided generator (P-Net) and an attention-based discriminator (G-Net) were jointly trained to refine segmentation masks with limited annotations. Their adversarial framework achieved a Dice score of 79.06% on the Abd-CT dataset. Similarly, Alsaleh et al. (2024) utilized the MAML technique and achieved mean Dice coefficients of 93.70% and 85.98% in 5-shot and 10-shot scenarios, respectively, using the TotalSegmentator dataset.

Despite their effectiveness, both generative and semi-supervised approaches still face several challenges. Generative models, such as GANs, often require substantial computational resources and high-quality labeled data to generate realistic synthetic samples. Semi-supervised learning methods, particularly those relying on pseudo-labeling or few-shot learning, often struggle with the quality and consistency of pseudo-labels, which can degrade performance when the model is exposed to noisy or ambiguous annotations. Moreover, these approaches often depend on large amounts of data to make up for the lack of labeled samples.

Moreover, existing methods face challenges with limited annotated data and domain adaptation. Hybrid neural networks and generative models often struggle with feature representation and computational efficiency. Attention-based approaches depend on large datasets, while semi-supervised methods require both labeled and unlabeled data. Many techniques also demand significant fine-tuning and computational resources. To address these challenges, we propose FSS-ULivR, a few-shot segmentation model that demonstrates robust performance across diverse datasets with minimal data.

## Method

This section details the architecture and components of our proposed FSS-ULivR model for few-shot liver segmentation. The model integrates a ResNet-based encoder with an enhanced prototype module that integrates a transformer block for global feature representation and an SE block for dynamic channel-wise refinement (Raiaan et al. 2024). In the decoder, attention gates are applied to effectively handle the challenges posed by limited annotated data (Rahman et al. 2022; Ouyang et al. 2022; Abian et al. 2024). Figure 2 demonstrates the overall structure of the FSS-ULivR model.

### Few-shot learning strategy

In our FSS-ULivR framework, our training follows a 1-shot episodic setup, where each episode provides exactly one support image–mask pair $(I_s, M_s)$ and a single query image $I_q$. The objective is to utilize this minimal supervision to predict the query mask $\hat{M}_q$ (Zhang et al. 2021). To achieve this, we first project both support and query images into a shared feature space using a deep encoder *E*, as demonstrated in Equation (1):

1 $$\begin{aligned} F_s, \{S_s^{(l)}\} = E(I_s), \quad F_q, \{S_q^{(l)}\} = E(I_q) \end{aligned}$$

where $F_* \in \mathbb {R}^{H \times W \times C}$ denotes the bottleneck feature map and $\{S_*^{(l)}\}$ are multi-scale feature maps used in skip connections. The encoder is composed of residual blocks, each incorporating a Squeeze-and-Excitation (SE) operation to recalibrate channel-wise activations. Next, we extract a refined prototype *P* from the support features using our Enhanced Prototype Module. After applying an SE block and a stack of transformer layers to $F_s$, we resize $M_s$ to match $F_s$ spatially and compute the prototype in Equation (2):

2 $$\begin{aligned} P = \frac{\sum _{i,j} F_s(i,j) \cdot M_s(i,j)}{\sum _{i,j} M_s(i,j) + \epsilon } \in \mathbb {R}^{1 \times 1 \times C} \end{aligned}$$

where $\epsilon$ is a small constant for numerical stability. We then apply safe L2 normalization and a channel-attention head to enhance the discriminative capacity of *P*. To localize the same semantic class in the query image, we refine $F_q$ through SE and transformer layers, and compute a similarity map by taking the dot-product between normalized query features and the prototype, which is expanded spatially to match the query features, as shown in Equation (3):

3 $$\begin{aligned} S_q(i,j) = \frac{\langle \tilde{F}_q(i,j), P \rangle }{\Vert \tilde{F}_q(i,j) \Vert \cdot \Vert P \Vert }, \quad S_q \in [0,1]^{H \times W \times 1} \end{aligned}$$

where $\tilde{F}_q$ denotes the normalized query feature map. The similarity map $S_q$ emphasizes regions likely to belong to the target class, and we modulate the original query features $F_q$ via element-wise multiplication of $F_q$ and $S_q$, which results in $F_q'$. The decoder, which mirrors the encoder using upsampling layers, Improved Attention Gates for each skip-connection, and residual blocks, takes as input $F_q'$ along with multi-scale skip features $S_q^{(l)}$ and reconstructs the output mask as demonstrated in Equation (4):

4 $$\begin{aligned} \hat{M}_q = \sigma \left( \text {Conv}_{1 \times 1} \left( D \left( \{F_q', S_q^{(l)}\} \right) \right) \right) \end{aligned}$$

where $D(\cdot )$ denotes the decoder module and $\sigma$ is the sigmoid activation function that produces the final binary mask.

The training objective optimizes a hybrid loss function that balances overlap, class imbalance, and boundary alignment by combining Dice loss, Focal loss, Tversky loss, and Binary Cross-Entropy (BCE) loss. This composite loss enables the model to effectively learn accurate segmentation boundaries while handling imbalanced data. By sampling diverse episodes with varying anatomical structures, FSS-ULivR generalizes effectively from just a single annotated support example.

### Encoder

Our encoder extracts high-level feature representations from both support and query images. It operates in two parallel branches (one for support and one for query) that share an identical architecture.

Each branch comprises multiple residual blocks enhanced with SE modules, and the features are progressively reduced by max pooling (Mahmud et al. 2021). This design preserves spatial hierarchies while maintaining rich feature representations that are crucial for subsequent fusion in the prototype module and refinement in the decoder.

**Fig. 3:**
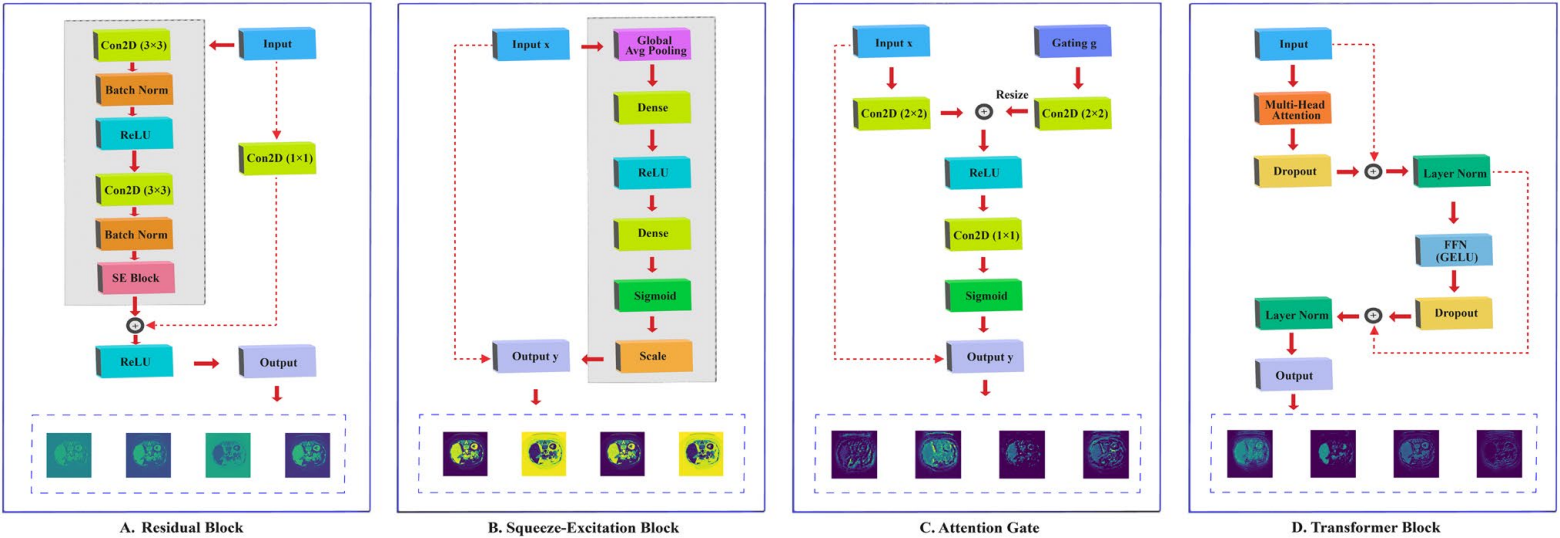
Illustration of the core components in the FSS-ULivR model: **A**. Residual Block, **B**. Squeeze-Excitation (SE) Block, **C**. Attention Gate, and **D**. Transformer Block, integrated for enhanced performance in few-shot segmentation

#### Residual blocks with SE modules

Residual blocks serve as the backbone of our encoder network. The input feature map $F_{\text {in}} \in \mathbb {R}^{C \times H \times W}$ is processed through the residual block, where $C$ represents the number of channels and $H \times W$ denote the spatial dimensions. Each block contains a main path with SE module, skip connections, and the output derived from the residual block. The integrated SE modules help the block focus on important channels and improve feature learning across different spatial levels. Figure 3A illustrates the Residual Block of our FSS-ULivR model.

We compute the main path first by applying a $3 \times 3$ convolution, then applying batch normalization and a Rectified Linear Unit (ReLU) activation as shown in Equation (5).

5 $$\begin{aligned} F_1 = \operatorname {ReLU}\Bigl ( \gamma \bigl (W_1 * F_{\text {in}} + b_1\bigr ) \Bigr ) \end{aligned}$$

where $W_1$ is a $3 \times 3$ kernel, $b_1$ is the bias, $\gamma$ denotes batch normalization, and $*$ represents the convolution operation. Then, we perform a second $3 \times 3$ convolution, after which batch normalization is applied in Equation (6).

6 $$\begin{aligned} F_2 = \gamma \Bigl (W_2 * F_1 + b_2\Bigr ) \end{aligned}$$

where $W_2$ is another $3 \times 3$ convolution kernel, and $b_2$ is its corresponding bias term. Following Equation (6), we refine the channel-wise features using an SE module, which includes squeeze, excitation, and recalibration steps. This refinement explicitly models interdependencies between channels, enhancing the representation of discriminative spatial hierarchies. Figure 3B illustrates the SE Block of our FSS-ULivR model.

The squeeze operation aggregates spatial information by computing channel-wise statistics through Global Average Pooling (GAP) in Equation (7), which results in a vector $z \in \mathbb {R}^{C}$.

7 $$\begin{aligned} z_c = \frac{1}{H \times W} \sum _{i=1}^{H} \sum _{j=1}^{W} F_2(c,i,j), \quad c = 1, \ldots, C \end{aligned}$$

The excitation operation then learns channel-specific scaling factors that capture the relative importance of each feature channel for the given spatial context. The vector $z$ is passed through two fully connected layers to generate these scaling factors in Equation (8):

8 $$\begin{aligned} s = \sigma \Bigl ( W_{se2} \cdot \operatorname {ReLU}\bigl (W_{se1} \cdot z + b_{se1}\bigr ) + b_{se2} \Bigr ) \end{aligned}$$

where $W_{se1} \in \mathbb {R}^{\frac{C}{r} \times C}$ and $W_{se2} \in \mathbb {R}^{C \times \frac{C}{r}}$ are weight matrices, $b_{se1}$ and $b_{se2}$ are biases, $r$ is the reduction ratio, and $\sigma$ refers to the sigmoid function. This two-stage fully connected architecture enables the SE module to learn complex, non-linear channel relationships while maintaining computational efficiency through dimensionality reduction. The recalibration step applies these learned scaling factors to selectively emphasize or suppress different channels based on their relevance to the current spatial context, as shown in Equation (9):

9 $$\begin{aligned} F_2^{\text {SE}}(c,i,j) = s_c \cdot F_2(c,i,j), \quad \forall \, c, i, j. \end{aligned}$$

This channel-wise recalibration is particularly beneficial for few-shot segmentation as it helps the encoder focus on the most discriminative features while preserving spatial hierarchies across different resolution levels, while also providing an adaptive reweighting mechanism that makes the residual blocks sensitive to task-specific variations in the support-query pairs, which is critical in few-shot scenarios. To facilitate gradient flow, we further add a skip connection, $F_{skip}$, and apply a $1 \times 1$ convolution for dimension matching before addition. The $F_{skip}$ is then passed as the input to the residual block $F_{in}$. The final output of the residual block $F_{\text {out}}$ is then obtained by integrating the recalibrated main path (see Equation (9)) with the skip connection, followed by a ReLU activation. Finally, we apply max pooling to reduce the spatial dimensions after each residual block and to capture contextual information at multiple scales.

#### Parallel processing for support and query

In our experiment, we processed both support and query images using identical encoder branches. Then we stored the intermediate feature maps at different levels as skip connections. These features in the decoder are used to recover spatial details lost during downsampling.

### Enhanced prototype module

Our Enhanced Prototype Module refines and fuses features from the support and query branches through two parallel processes. One processes support features to compute a robust prototype, while the other refines query features prior to fusion. The module utilizes transformer-based self-attention to capture global spatial dependencies and channel attention mechanisms to emphasize discriminative features, creating a unified approach that significantly improves support-query feature alignment in few-shot segmentation scenarios. In addition, we compute a cosine similarity map between the fused query features and the final support prototype by normalizing both along the channel dimension. This similarity map is then scaled to the range [0, 1] to form a spatial attention mask that guides the decoder.

#### Support processing stream

We first refine the support features using an SE block (as detailed in Equations (7)-(9)), ensuring that the most informative channels are emphasized. This channel-wise recalibration enhances the support features by emphasizing responses most relevant to the target class while suppressing background noise, which is particularly crucial when working with limited annotated examples. We then apply L2 normalization to these enhanced features before prototype pooling, ensuring that the subsequent similarity computation remains scale-invariant. We also refine the recalibrated support features utilizing transformer blocks as illustrated in Fig. 3D. The flattened support features are represented as $X \in \mathbb {R}^{N \times d}$, where $N$ denotes the number of tokens and $d$ refers to the feature dimension. We start the process by computing linear projections of query (*Q*), key (*K*), and value (*V*) for learnable parameter *X*. The transformer-based self-attention mechanism enables each spatial token in $X$ to aggregate information from all other tokens, capturing global anatomical and contextual relationships that conventional convolutional operations fail to represent effectively. We use normalization to keep the prototype and query features on a similar scale, making the cosine similarity calculation more accurate. We then compute scaled dot-product attention in Equation (10), and calculate multi-head attention (MHA) subsequently in Equation (11):

10 $$\begin{aligned} & \operatorname {Attention}(Q, K, V) = \operatorname {softmax}\left( \frac{QK^T}{\sqrt{d}} \right) V \end{aligned}$$

11 $$\begin{aligned} & \operatorname {MHA}(X) = \operatorname {Concat}\bigl (\operatorname {head}_1, \ldots, \operatorname {head}_h\bigr ) W_O \end{aligned}$$

where $W_O \in \mathbb {R}^{hd \times d}$ and $h$ is the number of heads. We then apply a feed-forward network (FFN) with subsequent residual connections and layer normalization in Equation (12):

12 $$\begin{aligned} \operatorname {FFN}(x) = W_{ff2} \cdot \operatorname {ReLU}\Bigl (W_{ff1} \cdot x + b_{ff1}\Bigr ) + b_{ff2} \end{aligned}$$

The refined support features are then passed through the residual connection, followed by layer normalization in 2 steps. In the first step, we compute the output of the MHA, $X'$, to the original input *X*. Then we applied layer normalization to this sum. In the second step, we add this to the output of the FFN, followed by second-layer normalization to generate $X''$. The refined support features preserve both the original context and the newly captured dependencies. This global context modeling produces features that are robust to local variations in shape, size, or appearance, which is essential, particularly when only a few annotated examples are available for training. We then compute the support prototype using a binary support mask $M \in \{0,1\}^{H \times W}$, which aggregates the features corresponding to the target class into a compact representation as demonstrated in Equation (13):

13 $$\begin{aligned} P = \frac{\sum _{i,j} M(i,j) \cdot X''(i,j)}{\sum _{i,j} M(i,j) + \epsilon } \end{aligned}$$

where $\epsilon$ represents a minimal value to avoid division by zero. Here $X''$ has already been L2-normalized in its channel dimension, so $P$ inherits normalized scale. We further refine the prototype by applying a channel attention mechanism as shown in Equation (14):

14 $$\begin{aligned} P_{\text {attn}} = \sigma \Bigl ( W_{\text {attn2}} \cdot \operatorname {ReLU}\bigl (W_{\text {attn1}} \cdot P + b_{\text {attn1}}\bigr ) + b_{\text {attn2}} \Bigr ) \odot P \end{aligned}$$

where $\odot$ denotes the element-wise multiplication. This channel attention mechanism adaptively adjusts the prototype based on channel-wise relevance, producing a support prototype $P_{\text {attn}}$ that is both semantically rich from global context modeling and refined through attention-based weighting of feature channels.

#### Query processing stream

We first recalibrate the query features, denoted by $X_{\text {query}}$, using an SE block, and then refine them by transformer blocks similar to those used in the support stream. The SE block enhances query features by emphasizing channels that correspond to the support prototype’s characteristics, while the transformer blocks provide each query location with global context, improving detection of target objects despite appearance variations. To ensure accurate comparison, we first L2-normalize the query feature vectors along the channel dimension so they match the scale of the support prototype before computing similarity. Specifically, we capture long-range dependencies in our query features by utilizing MHA, which is subsequently refined using FFN. The recalibrated query features, $X_{\text {query}}'$, are computed after processing the first recalibrated features with an MHA followed by a normalization layer; it is then refined (denoted as $X_{\text {query}}''$) with an FFN followed by a second normalized layer. We then fuse the refined query features with the support prototype to refine the query features by utilizing information from the support set. To align the spatial dimensions, we broadcast the support prototype $P$ to form $\tilde{P}$. Our fusion operation is defined in Equation (15):

15 $$\begin{aligned} F_{\text {fused}} = \operatorname {ReLU}\Bigl ( \gamma \Bigl (W_{\text {fuse}} * \operatorname {Concat}\bigl (X_{\text {query}}'', \tilde{P}\bigr ) + b_{\text {fuse}} \Bigr ) \Bigr ) \end{aligned}$$

where $\operatorname {Concat}$ combines the query features with the broadcast support prototype. Then we compute a similarity map based on the fused features and the channel-modulated support prototype in Equation (16):

16 $$\begin{aligned} S(i,j) = \frac{\langle F_{\text {fused}}(i,j), P_{\text {attn}} \rangle }{\Vert F_{\text {fused}}(i,j)\Vert \, \Vert P_{\text {attn}}\Vert } \end{aligned}$$

Both vectors are L2-normalized along the channel dimension, so $S(i,j)$ ranges between $-1$ and $1$. We then scale it to the range $[0,1]$ using $\frac{S + 1}{2}$ and clip values to avoid numerical instability. We then refine the query features to construct modulated features $F_{mod}$, using the similarity score, and scaling the fused features, $F_{fused}$, based on their similarity to the support prototype, *S*(*i*, *j*). This similarity map acts as an attention mask, highlighting regions most similar to $P_{\text {attn}}$ and filtering out irrelevant areas. By integrating spatially global self-attention with channel-wise recalibration in both support and query streams, the Enhanced Prototype Module achieves precise and context-aware support-query feature alignment, thereby significantly boosting few-shot segmentation accuracy and robustness under limited annotation scenarios. The resulting $F_{\text {mod}}$ is then passed to the decoder, so that the following upsampling and fusion with skip connections focus on areas most likely to contain the target class.

### Decoder

Our decoder reconstructs the segmentation map from the modulated query features by incorporating high-resolution details from the encoder’s skip connections, following a design with progressive upsampling, attention gating, and residual refinement (Li et al. 2024). The integration of improved attention gates and residual refinement blocks provides crucial advantages for restoring fine spatial details, particularly in recovering object boundaries and small-scale structures essential for accurate few-shot segmentation.

#### Upsampling and skip connections

At each decoding level $l$, we upsample the modulated feature map $F_{\text {mod}}^{(l)}$ to match the spatial dimensions of the corresponding encoder feature map $F_{\text {enc}}^{(l)}$, as $F_{\text {up}}^{(l)}$, where each of the following modulated feature map is upsampled. We then concatenate the upsampled feature with the encoder feature. This concatenation $F_{cat}^{(l)}$ allows us to recover spatial details that were lost during downsampling.

#### Improved attention gates

We apply attention gates as illustrated in Fig. 3C to the skip connections to emphasize the most relevant features. These gates address the fundamental challenge of selective feature integration by dynamically weighting skip connections based on spatial and contextual relevance, effectively suppressing background noise while preserving target object boundaries. We then apply $1 \times 1$ convolutions to the skip connection feature $x$ and the gating signal $g$ from the upsampled features, as described in Equation (17):

17 $$\begin{aligned} \tilde{x} = W_x * x + b_x, \quad \tilde{g} = W_g * g + b_g. \end{aligned}$$

We then fuse these two transformed features and apply a ReLU activation to generate the attention coefficients, as $f$. Next, we compute the attention map $\alpha$ using a $1 \times 1$ convolution and then applying a sigmoid activation. We then modulate the skip connection ($x_{attn}$) by combining the initial skip connection feature *x* with the attention map $\alpha$, which emphasizes the relevant spatial features to preserve fine-grained details while suppressing irrelevant ones.

#### Feature fusion and residual refinement

In this stage, we concatenate the modulated skip connection $x_{\text {attn}}$ with the upsampled feature map $F_{\text {up}}^{(l)}$ for creating the fused feature set $F_{\text {fused}}^{(l)}$. This fused feature is then refined using additional residual blocks with SE modules, following the same formulation as described in Sect. 3.2.1. The residual refinement blocks help improve training by ensuring effective gradient flow and progressively enhancing features through channel-wise recalibration with SE modules. This process is crucial for recovering fine spatial details, as the SE modules adaptively emphasize important channels that capture object boundaries and small structures, while the residual connections preserve key spatial information throughout refinement. After the final refinement, we apply a convolutional layer to produce the segmentation logits as $F_{\text {logits}}$. Finally, we use a sigmoid activation function ($\sigma$) to convert the logits $F_{logits}(i, j)$ into probabilities, which generates the final segmentation mask $\hat{y}(i,j)$. By selectively emphasizing relevant features and adaptively recalibrating channels, the attention gates and residual refinement blocks with SE modules enable precise restoration of fine details, which is crucial for accurate few-shot segmentation from limited data.

### Loss functions

The FSS-ULivR model employs a composite loss function that integrates multiple loss metrics, including Dice loss, Focal loss, Tversky loss, and Binary Cross-Entropy (BCE) loss to improve segmentation accuracy and robustness. The Dice loss is derived from the Dice coefficient (see Sect. 4.2.1). The BCE loss, $L_{\text {BCE}}$, is given by Equation (18):

18 $$\begin{aligned} L_{\text {BCE}} = -\frac{1}{N} \sum _{i=1}^N \left[ y_i \log \hat{y}_i + (1 - y_i) \log (1 - \hat{y}_i) \right] \end{aligned}$$

where $y_i$ and $\hat{y}_i$ represent the true label and predicted probability for the $i$-th pixel, respectively. The Focal loss, $L_{\text {Focal}}$ as defined in Equation (19), addresses class imbalance by reducing the relative loss for well-classified examples and focusing more on hard, misclassified examples.

19 $$\begin{aligned} \begin{aligned} L_{\text {Focal}}&= - \frac{1}{N} \sum _{i=1}^N \alpha \,(1 - p_i)^\gamma \bigl [\,y_i \log (p_i) \\&\qquad \quad + (1 - y_i)\log (1 - p_i)\bigr ] \end{aligned} \end{aligned}$$

where $p_i = \hat{y}_i$ is the predicted probability for class 1, $\alpha$ is a weighting factor for balancing positive and negative examples, and $\gamma$ is the focusing parameter that adjusts the rate at which easy examples are down-weighted. The Tversky loss, $L_{\text {Tversky}}$, is a generalization of the Dice loss that allows control over the penalty of false positives and false negatives as defined in Equation (20), which is useful for imbalanced datasets:

20 $$\begin{aligned} L_{\text {Tversky}} = 1 - \frac{TP + \epsilon }{TP + \alpha \, FN + \beta \, FP + \epsilon } \end{aligned}$$

where $TP$, $FN$, and $FP$ denote true positives, false negatives, and false positives, respectively; $\alpha$ and $\beta$ control the penalties for false negatives and false positives; and $\epsilon$ is a small constant to avoid division by zero. The final combined loss function, computed in Equation (21), is a weighted sum of these individual losses.

21 $$\begin{aligned} L_{\text {combined}} = \alpha \, L_{\text {Dice}} + \beta \, L_{\text {Focal}} + \gamma \, L_{\text {Tversky}} + \delta \, L_{\text {BCE}} \end{aligned}$$

where $\alpha$, $\beta$, $\gamma$, and $\delta$ are non-negative weighting coefficients balancing the contributions of each loss term, with their sum equal to one. This integrated loss formulation ensures both accurate pixel-level boundary delineation and effective handling of class imbalance, leading to improved segmentation performance. The values of these coefficients were empirically determined. Further details of this tuning process are discussed in Sect. 5.6.

### Training procedure

Our training procedure consists of two stages: episodic few-shot segmentation training on the LiTS dataset, followed by cross-dataset validation on multiple external datasets.

In the first stage, we create 1-shot episodes by sampling a single support image–mask pair $(I_s, M_s)$ and a query image $I_q$ from the LiTS training set. Each raw CT slice is first min–max normalized, resized to the target resolution, and windowed to highlight soft-tissue regions.

The processed support and query images are then passed through our ResNet-based encoder with SE blocks to extract multi-scale features. The Enhanced Prototype Module computes a refined support prototype and creates a similarity map, which is used to guide the query features during decoding. We train the model using a combined loss function composed of Dice loss, Focal loss, Tversky loss, and Binary Cross-Entropy (BCE) loss. The training employs the Adam optimizer with a fixed learning rate of 0.001, gradient clipping with a norm of 1.0, and a batch size of one episode. After completing *N* epochs of training, we freeze the model parameters and perform cross-dataset validation. For each external dataset $D_i \in \{\text {3DIRCADB01}, \text {CRLM}, \text {CT-ORG},$ MSD-Task03-Liver}, we prepare the data loader and normalization steps, then evaluate the trained model on all available images from that dataset.

We compute metrics such as Dice coefficient, specificity, and IoU to measure the model’s performance. No additional training or fine-tuning is applied during this evaluation; we directly test the trained model to show how well FSS-ULivR works on data with different characteristics and from different sources.

Algorithm 1 provides an overview of both the episodic training and the evaluation process. This two-stage approach ensures that our model not only learns from limited examples but also performs well on entirely new datasets without needing extra adjustments.

**Algorithm 1:**
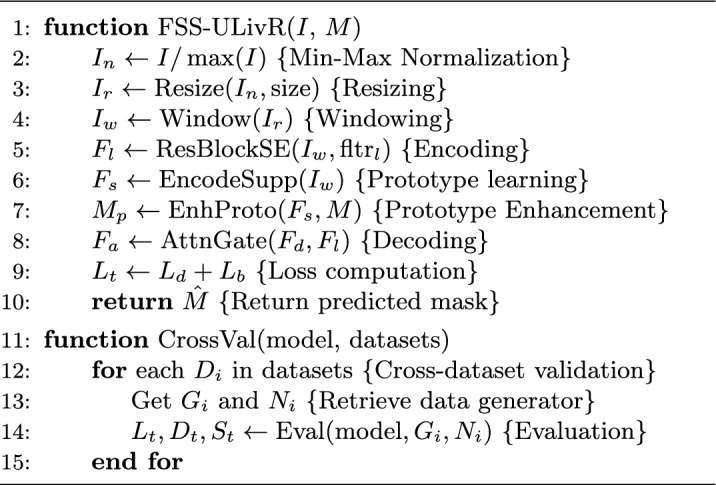
FSS-ULivR: Few-Shot Segmentation Workflow

## Experimental details

This section outlines the experimental setup for performance analysis of our proposed FSS-ULivR model, including data preparation and preprocessing steps, evaluation metrics, and implementation details.

### Data preparation

#### Dataset

We utilized seven unique datasets for evaluating the performance of our few-shot segmentation model. Among the datasets, the LiTS^1^ dataset has been used for training, while 3D-IRCADb-01,^2^ CRLM,^3^ CT-ORG,^4^ and MSD-Task03-Liver,^5^ BraTS 2019,^6^ and BraTS 2020^7^ datasets were employed for cross-dataset validation purposes. Table 1 summarizes the sample distribution.

**Table 1:** Distribution of samples across datasets

Datasets	# Subjects	Modality	Purpose
LiTS (Bilic et al. 2023)	130	CT	Train, Validation & Test
3D-IRCADb-01 (Soler et al. 2015)	20	CT	Cross-Dataset Validation
CRLM (Simpson et al. 2024)	197	CT	Cross-Dataset Validation
CT-ORG (Rister et al. 2019)	140	CT	Cross-Dataset Validation
MSD-Task03-Liver (Antonelli et al. 2022)	123	CT	Cross-Dataset Validation
BraTS 2019 (Bakas et al. 2017)	335	MRI	Cross-Dataset Validation
BraTS 2020 (Menze et al. 2014)	494	MRI	Cross-Dataset Validation

*Liver Tumor Segmentation Challenge (LiTS).* The LiTS dataset (Bilic et al. 2023) comprises 130 volumetric abdominal Computed Tomography (CT) scans, each with a pixel resolution of $512 \times 512$ and varying voxel spacings, with in-plane resolutions between 0.60 and 0.98 mm and slice thicknesses ranging from 0.45 to 5.0 mm. It includes a range of liver-related abnormalities such as tumors, metastases, and cysts. Tumor volumes vary from 38 mm^3^ to 1231 mm^3^, and the number of tumors per scan ranges from 0 to 12.

*3D-IRCADb-01.* The 3D-IRCADb-01 dataset (Soler et al. 2015) comprises 3D CT scans from 20 patients, featuring diverse cases with tumor counts ranging from 0 to 75 per scan and tumor sizes varying from 38 mm³ to 349 cm³. The CT scans have slice thicknesses varying from 0.625 mm to 2.5 mm. Despite the limited number of subjects, each scan provides a rich volume of spatial information, along with high-quality expert annotations and substantial variation in tumor type, size, and location, making it a valuable benchmark for assessing model generalization, particularly in few-shot and cross-dataset evaluation scenarios.

*Colorectal Liver Metastases (CRLM).* The CRLM dataset (Simpson et al. 2024) includes preoperative contrast-enhanced 3D CT scans from 197 patients who underwent resection of colorectal liver metastases. The scans were collected using a multidetector CT scanner with autoMA 220–380, a noise index between 12 and 14, a rotation time of 0.7–0.8 ms, and a scan delay of 80 s.

*Computed Tomography Organs (CT-ORG).* The CT-ORG dataset (Rister et al. 2019) consists of 140 CT scans along with 3D annotations for five organs, including lungs, bones, liver, kidneys, and bladder. A subset also includes brain annotations. The dataset includes diverse imaging conditions, with 131 dedicated CTs and 9 PET-CT components, making it suitable for multi-organ segmentation tasks.

*Medical Segmentation Decathlon Challenge Task 3: liver (MSD-Task03-Liver).* The MSD-Task03-Liver dataset (Antonelli et al. 2022) consists of a collection of CT and MRI scans for liver and tumor segmentation. It includes 123 samples of 3-dimensional volumetric data with an average image dimension of approximately $205\times 205\times 160$ voxels.

*Brain Tumor Segmentation Challenge 2019 (BraTS 2019).* The BraTS 2019 dataset (Bakas et al. 2017) comprises 335 multi-institutional, pre-operative multimodal MRI scans of patients with glioblastoma (GBM) and lower grade glioma (LGG), along with pathologically confirmed diagnoses and overall survival (OS) data. It includes four MRI modalities: native T1, post-contrast T1 (T1Gd), T2, and FLAIR. Expert neuroradiologists manually segmented the tumor subregions, including the enhancing tumor, peritumoral edema, and necrotic or non-enhancing core. All scans are skull-stripped, co-registered to a standard anatomical template, and resampled to 1 mm$^3$ isotropic resolution.

*Brain Tumor Segmentation Challenge 2020 (BraTS 2020).* The BraTS 2020 dataset (Menze et al. 2014) is widely used for brain tumor segmentation and classification tasks. It comprises four MRI modalities: Fluid Attenuated Inversion Recovery (FLAIR), T1-weighted (T1), T2-weighted (T2), and contrast-enhanced T1-weighted (T1-CE) images. The training set includes 369 subjects, and the validation set contains 125 subjects, with each volumetric MRI scan having spatial dimensions of $240\times 240\times 155$, where 155 represents the number of axial slices.

#### Data preprocessing

In this subsection, we outline the preprocessing techniques applied to standardize and enhance the input data for segmentation. The preprocessing pipeline begins with Min-Max normalization to adjust intensity variations across the images, followed by resizing the images to a consistent 256$\times$256 pixel resolution. Finally, a windowing operation is applied to highlight the relevant tissues. A concise overview of these preprocessing steps is provided in Algorithm 2.

**Algorithm 2:**
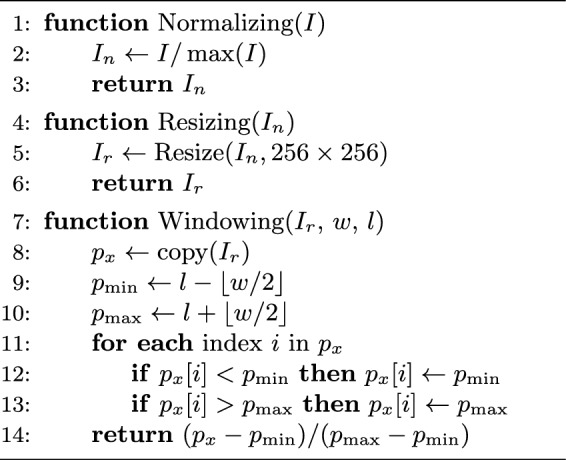
Preprocessing Pipeline for FSS-ULivR

### Evaluation metrics

For evaluating the FSS-ULivR model on liver segmentation tasks, we use the Dice coefficient, specificity metrics, and combined loss functions, which together assess the overlap between predicted and ground truth segmentations while addressing class imbalance effectively (Rahman et al. 2025; Abian et al. 2025; Eelbode et al. 2020).

#### Dice coefficient

The Dice Coefficient measures the overlap between the predicted and ground truth masks, as shown in Equation (22):

22 $$\begin{aligned} \textrm{Dice} = \frac{2 \times \sum _{i=1}^{N} (p_i \cdot y_i)}{\sum _{i=1}^{N} p_i + \sum _{i=1}^{N} y_i} \end{aligned}$$

where $N$ represents the total number of pixels, $p_i$ is the predicted mask, and $y_i$ denotes the ground truth mask.

#### Intersection over Union (IoU)

The Intersection over Union (IoU), also known as the Jaccard Index, measures the overlap between the predicted and ground truth masks relative to their union. It is computed as shown in Equation (23):

23 $$\begin{aligned} \textrm{IoU} = \frac{\sum _{i=1}^{N} (p_i \cdot y_i)}{\sum _{i=1}^{N} p_i + \sum _{i=1}^{N} y_i - \sum _{i=1}^{N} (p_i \cdot y_i)} \end{aligned}$$

where $N$ represents the total number of pixels, $p_i$ is the predicted mask, and $y_i$ denotes the ground truth mask.

#### Specificity

Specificity represents the ratio of true negatives over the sum of true negatives and false positives, indicating how well the model avoids incorrectly labeling negative cases as positive. It is calculated as shown in Equation (24):

24 $$\begin{aligned} \text {Specificity} = \frac{\text {True Negatives}}{\text {True Negatives} + \text {False Positives}} \end{aligned}$$

### Implementation details

This research is conducted using an AMD Ryzen 5 5600X 6-core Central Processing Unit (CPU) and 16 GB of RAM for all experiments. For graphical processing, a ZOTAC GAMING GeForce RTX 3060 Twin Edge OC with 12 GB of video RAM (VRAM) is utilized, while Jupyter Notebook version 7.0.8 serves as the integrated development environment (IDE).

## Results

In this section, we present the performance of FSS-ULivR across multiple liver segmentation benchmarks, evaluating Dice coefficient, IoU, and specificity. Results on both in-domain and cross-dataset settings demonstrate the model’s high accuracy, robustness, and generalizability, even with limited annotated data.

### Qualitative performance assessment

#### Segmentation performance of the proposed FSS-ULivR model

To assess the segmentation performance of the proposed FSS-ULivR model, we evaluated it on the LiTS test dataset using uniformly preprocessed slices of size $256 \times 256$. The model was trained with the Adam optimizer at a learning rate of 0.001 for 200 epochs under the 1-shot episodic setup.

**Fig. 4:**
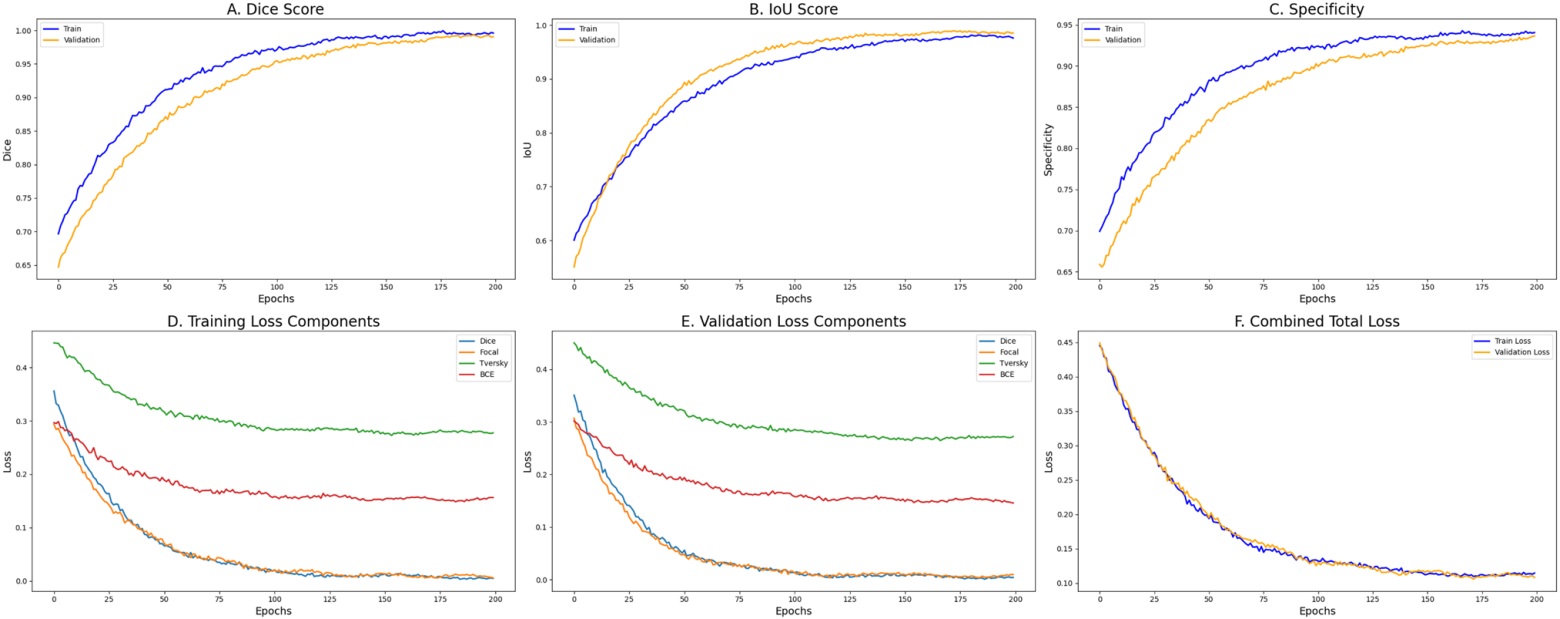
Training and validation curves for our proposed FSS-ULivR model

**Table 2:** Overall segmentation performance of FSS-ULivR on the LiTS dataset

Metric	Performance
Dice Similarity Coefficient (DSC)	98.94 ± 0.24%
Intersection over Union (IoU)	97.44 ± 1.09%
Specificity	93.78 ± 3.72%
Test Loss	0.0735

**Fig. 5:**
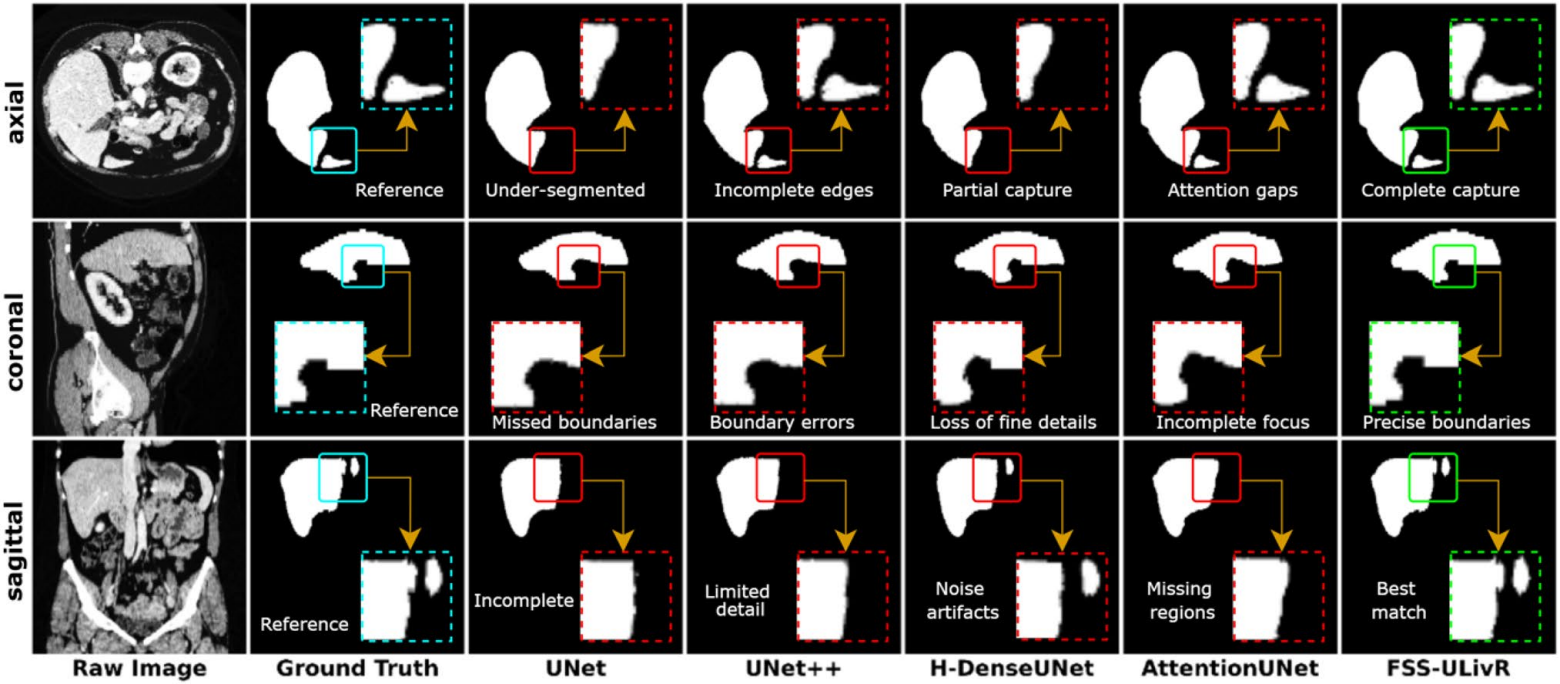
Comparative performance of the proposed FSS-ULivR model with U-Net, U-Net++, H-DenseUNet, and AttentionUNet

As shown in Table 2, FSS-ULivR achieves a Dice coefficient of 98.94±0.24%, an IoU of 97.44±1.09%, and a specificity of 93.78±3.72% on the test set, with a test loss of 0.0735, which combines Dice, Focal, Tversky, and BCE losses. These results demonstrate that our prototype-enhanced attention mechanisms and residual refinement-based few-shot segmentation model achieved highly accurate liver segmentation with minimal supervision.

#### Training progress and convergence

The training and validation curves (Sutradhar et al. 2025) of our proposed FSS-ULivR model demonstrate stable convergence and robust segmentation performance across 200 training epochs.

As illustrated in Fig. 4A, the Dice coefficient steadily increases for both training and validation, ultimately reaching 99.91% and 99.94%, respectively, indicating highly accurate segmentation results with minimal fluctuations. Similarly, Intersection over Union (IoU) scores, shown in Fig. 4B, increase progressively throughout training, achieving values of 98.53% for training and 98.94% for validation, indicating excellent overlap between predicted and ground truth masks.

Specificity metrics in Fig. 4C further highlight the ability of the proposed FSS-ULivR model to correctly identify negative pixels, with values consistently approaching 94% across both training and validation, reflecting effective distinction between foreground and background regions. The component-wise loss plots in Fig. 4D and E illustrate the progressive minimization of all loss functions, including Dice loss, Focal loss, Tversky loss, and Binary Cross-Entropy (BCE) loss during training and validation, respectively, demonstrating stable optimization. Figure 4F presents the combined total loss for both training and validation, showing a smooth and consistent decrease throughout training, ultimately stabilizing around 0.1109 for training and 0.1075 for validation. These observations validate the efficacy of our prototype-guided few-shot segmentation strategy, enhanced by attention and residual refinement modules, enabling the model to converge efficiently and provide high accuracy with limited annotated data.

### Comparison with state-of-the-art methods

The performance of the proposed FSS-ULivR model was assessed against several state-of-the-art (SOTA) segmentation models, including U-Net (Ronneberger et al. 2015), U-Net++ (Zhou et al. 2018), H-DenseUNet (Li et al. 2018), and AttentionUNet (Oktay et al. 2018). All models were evaluated using a uniform image dimension of $256 \times 256$ pixels and trained with the Adam optimizer under consistent training settings of 200 epochs, a learning rate of 0.001. The FSS-ULivR model achieved the highest performance with a Dice coefficient of 98.94±0.24%, an Intersection over Union (IoU) of 97.44±1.09%, and a specificity of 93.78±3.72%. In comparison, the U-Net model yielded a Dice coefficient of 95.63±1.03%, IoU of 91.98±0.71%, and specificity of 85.29±2.94%. U-Net++ improved the Dice score to 96.86±2.41%, IoU to 92.15±1.30%, and specificity to 87.65±3.06%. H-DenseUNet reached a Dice coefficient of 97.25±0.91%, IoU of 93.59±1.16%, and specificity of 89.01±1.94%, while AttentionUNet attained a Dice coefficient of 97.46±0.58%, IoU of 94.08±0.88%, and specificity of 92.88±2.61%. Overall, the segmentation models showed Dice coefficients ranging from approximately 95.6% to 97.5%, IoU values between 92% and 94.1% and specificity values between 85.3% and 92.9%, clearly demonstrating the superior performance of the FSS-ULivR model in accurately segmenting target regions. Table 3 reports the model’s comparative performance against the SOTA methods.

**Table 3:** Comparative performance of the proposed FSS-ULivR with U-Net-based state-of-the-art models in Dice Similarity Coefficient (DSC%), Intersection over Union (IoU%), and Specificity (Spec%)

Model	DSC (%)	IoU (%)	Spec. (%)
H-DenseUNet (Li et al. 2018)	97.25 ± 0.91	93.59 ± 1.16	89.01 ± 1.94
U-Net (Ronneberger et al. 2015)	95.63 ± 1.03	91.98 ± 0.71	85.29 ± 2.94
U-Net++ (Zhou et al. 2018)	96.86 ± 2.41	92.15 ± 1.30	87.65 ± 3.06
Attention U-Net (Oktay et al. 2018)	97.46 ± 0.58	94.08 ± 0.88	92.88 ± 2.61
FSS-ULivR (Ours)	**98.94 ± 0.24**	**97.44 ± 1.09**	**93.78 ± 3.72**

Bold text represents the highest results

We further compared our model with several recent state-of-the-art Segment-Anything Models to show the proposed model’s effectiveness (see Table 4) on the LiTS dataset. Our model exceeded these models in terms of Dice score and achieved the highest Dice score of 98.94±0.24%. Figure 5 visualizes the semantic segmentation capabilities of our model compared to the SOTA methods.

**Table 4:** Comparative performance of the proposed model with state-of-the-art SAM-based models on the LiTS dataset in terms of Dice Similarity Coefficient (DSC)

Ref.	Model	DSC (%)
Chen et al. (2024)	ASLseg	74.28±0.27
Shi et al. (2024)	M-SAM	89.95±0.00
Wu et al. (2025)	Med-SA	83.67±0.00
Wang et al. (2025)	SAM-Med3D	88.71±0.00
Bui et al. (2024)	SAM3D	82.27±0.00
	FSS-ULivR (Our Study)	**98.94±0.24**

Bold text represents the highest results

### Comparison with existing literature

Table 5 provides a comparison of liver segmentation performance on the LiTS dataset, where several existing methods are compared using the Dice coefficient metric.

**Table 5:** Performance comparison between existing literature and our proposed FSS-ULivR model on the LiTS dataset

Ref.	Model	Dice-coefficient (%)
Li et al. (2018)	H-DenseUNet	98.2±1.00
Liu et al. (2023)	PCGAN	71.4±16.2
Hettihewa et al. (2023)	MANet	81.45±0.15
Seo et al. (2019)	mU-Net	98.51±1.02
Lei et al. (2021)	DefED-Net	96.30±1.01
Meng et al. (2020)	TDP-CNN	94.60±0.00
Zhang et al. (2022)	DPC-Net	96.00±0.00
Chen et al. (2024)	ASLseg	74.28±0.27
	FSS-ULivR (Our Study)	**98**.**94**±**0**.**24**

Bold text represents the highest results

Liu et al. (2023) employed a GAN-based approach (PCGAN) and obtained a Dice score of 71.4%. On the other hand, Chen et al. (2024) achieved a Dice score of 74.28±0.27% with their ASLseg method, while Hettihewa et al. (2023) obtained a Dice score of 81.45±0.15% with their MANet method. Meng et al. (2020) achieved 94.6% with the TDP-CNN model. Li et al. (2018) achieved a Dice score of 98.2% with H-DenseUNet, while Lei et al. (2021) obtained 96.30±1.01% with DefED-Net. Seo et al. (2019) achieved one of the highest Dice scores with mU-Net, reaching 98.51±1.02%, which outperformed Zhang et al. (2022) with DPC-Net (96%). Additionally, a few studies have explored few-shot learning strategies for liver segmentation. For example, Kim et al. (2021) introduced a 3D few-shot segmentation model (FSSBiGRU) utilizing a bidirectional GRU to enforce consistency across adjacent CT slices, combined with transfer learning for organ adaptation. Their approach achieved a Dice score of 88.7% on the BCV dataset. Awudong et al. (2024) proposed PG-Net, a prototype-based generative adversarial network, which jointly trains a prototype-guided generator and an attention-based discriminator to refine segmentation masks with limited annotations. This method attained a Dice score of 79.06% on the Abd-CT dataset. Moreover, Alsaleh et al. (2024) applied a Model-Agnostic Meta-Learning (MAML) framework, achieving mean Dice coefficients of 93.70% and 85.98% in 5-shot and 10-shot settings, respectively, on the TotalSegmentator dataset. In comparison, our proposed FSS-ULivR method achieves a Dice score of 98.94±0.24%, surpassing all existing few-shot and conventional methods, demonstrating the superior segmentation performance of our approach.

**Table 6:** Cross-dataset evaluation of the proposed FSS-ULivR Compared to U-Net, U-Net++, TransUNet, and Swin-Unet models on 3DIRCADB01, CRLM, CT-ORG, and MSD-Task03-Liver datasets using Dice coefficient, Intersection over Union (IoU), and Specificity

Dataset	Metrics (%)	U-Net (Ronneberger et al. 2015)	U-Net++ (Zhou et al. 2018)	TransUNet (Chen et al. 2024)	Swin-Unet (Cao et al. 2022)	FSS-ULivR
3DIRCADB01	Dice	92.35±2.91	93.01±1.52	93.88±0.86	94.23±1.07	**95.43±1.32**
	IoU	89.12±3.07	90.04±2.94	91.05±3.19	91.67±2.76	**92.20±1.75**
	Specificity	90.28±1.89	91.63±0.91	92.15±1.54	92.86±2.01	**93.81±0.73**
CRLM	Dice	89.75±2.68	90.42±1.37	91.03±0.49	91.55±1.16	**92.98±1.83**
	IoU	86.27±2.79	87.21±3.01	88.09±2.94	89.01±2.80	**90.16±2.08**
	Specificity	93.16±1.51	94.11±1.01	94.76±1.71	95.08±2.61	**95.93±1.54**
CT-ORG	Dice	87.62±2.47	88.39±1.56	89.12±0.77	90.04±1.10	**90.72±1.69**
	IoU	83.13±3.58	84.28±2.94	85.02±3.34	**86.52±3.60**	86.47±2.06
	Specificity	92.34±2.01	93.17±1.94	93.62±2.21	94.18±1.88	**94.85±1.37**
MSD-Task03-Liver	Dice	91.32±2.86	92.04±1.34	92.66±0.61	93.17±1.17	**94.05±0.73**
	IoU	86.92±3.09	87.88±2.90	88.54±3.11	89.01±1.89	**89.32±3.37**
	Specificity	89.67±0.97	90.41±1.34	90.93±1.61	91.28±2.00	**91.53±1.66**

Bold text represents the highest results

**Fig. 6:**
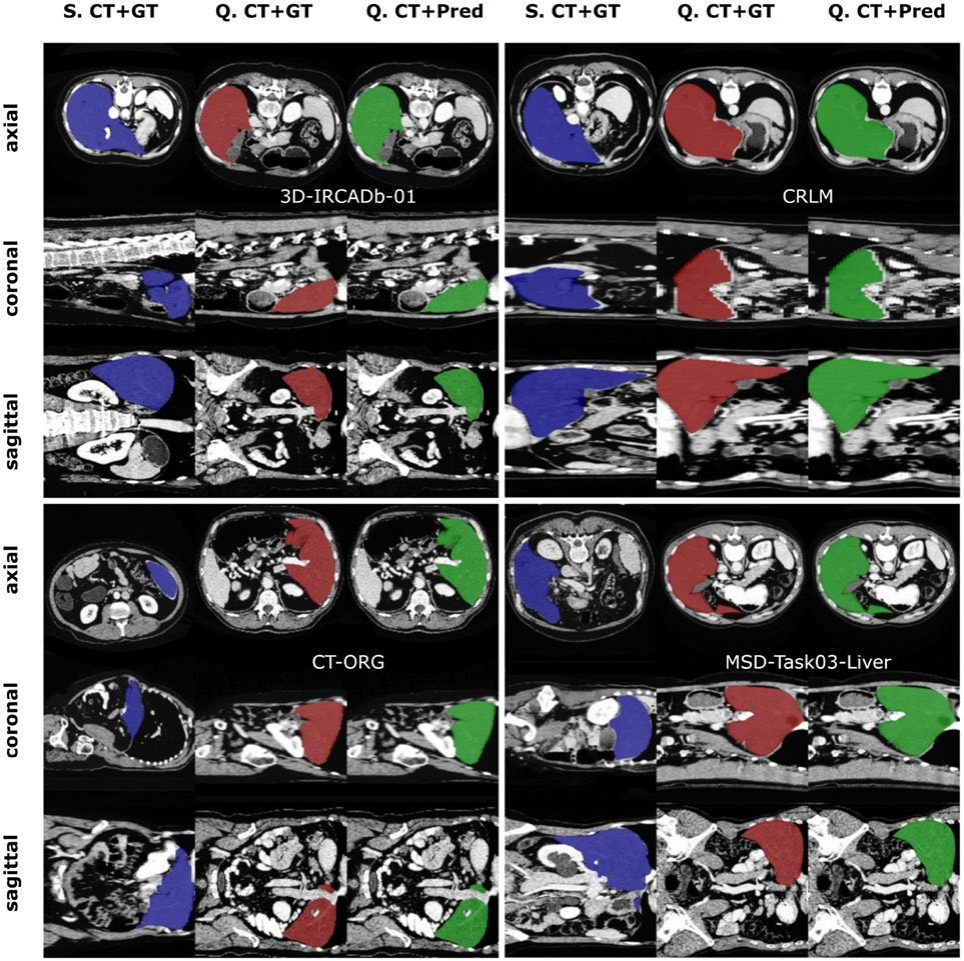
The cross-dataset evaluation results demonstrate the FSS-ULivR model’s performance on the 3Dircadb01, CRLM, CT-ORG, and MSD-Task03-Liver datasets. Each row displays different anatomical views (axial, coronal, and sagittal) with columns representing S (Support image), Q (Query image), GT (Ground Truth segmentation), and Pred (Predicted segmentation)

**Fig. 7:**
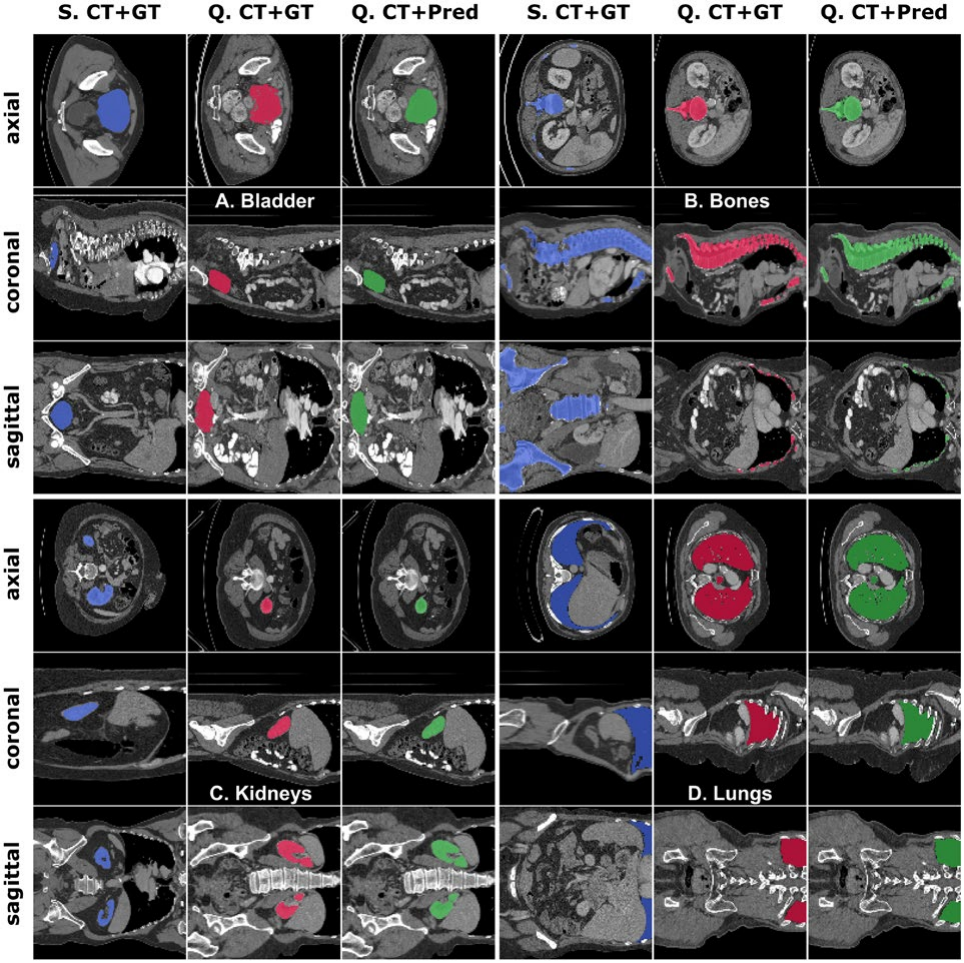
Multi-organ segmentation results of FSS-ULivR model on the CT-ORG dataset, demonstrating accurate delineation of **A**. Bladder, **B**. Bones, **C**. Kidneys, and **D**. Lungs across different anatomical structures. Each row displays different anatomical views (axial, coronal, and sagittal) with columns representing S (Support image), Q (Query image), GT (Ground Truth segmentation), and Pred (Predicted segmentation)

### Cross dataset evaluation

To assess the robustness and generalization capabilities of the proposed FSS-ULivR model, we conducted a cross-dataset evaluation across four external datasets, including 3DIRCADB01, CRLM, CT-ORG, and MSD-Task03-Liver. These datasets cover various clinical scenarios and liver segmentation challenges, enabling a detailed assessment of the performance and reliability of the model. The trained FSS-ULivR model, which was originally optimized on the LiTS Dataset, was evaluated using the same architecture, image size of 256$\times$256 dimensions, with Adam optimizer, learning rate of 0.001, with 200 epochs. It significantly outperformed other SOTA methods, including U-Net (Ronneberger et al. 2015), U-Net++ (Zhou et al. 2018), TransUNet (Chen et al. 2024), and Swin-Unet (Cao et al. 2022), across all external datasets, demonstrating its exceptional generalization and robustness. Specifically, on the 3DIRCADB01 dataset, FSS-ULivR achieved a Dice coefficient of 95.43±1.32%, an IoU of 92.20±1.75%, and a specificity of 93.81±0.73%, outperforming other SOTA models in accurately delineating liver structures. In the CRLM dataset, FSS-ULivR obtained a Dice coefficient of 92.98±1.83%, an IoU of 90.16±2.08%, and a specificity of 95.93±1.54%, effectively handling the complexities inherent in liver segmentation. The CT-ORG dataset further confirmed the superior performance of FSS-ULivR with a Dice coefficient of 90.72±1.69%, an IoU of 86.47±2.06%, and a specificity of 94.85±1.37%, even when dealing with varying acquisition protocols. Lastly, on the MSD-Task03-Liver dataset, FSS-ULivR achieved a Dice coefficient of 94.05±0.73%, an IoU of 89.32±3.37%, and a specificity of 91.53±1.66%, further highlighting its reliability and potential for clinical applications. Table 6 summarizes the performance metrics, clearly illustrating that FSS-ULivR outperforms U-Net, U-Net++, TransUNet, and Swin-Unet in both Dice coefficient and specificity across all evaluated datasets, and Fig. 6 demonstrates the performance of the FSS-ULivR model on the 3Dircadb01, CRLM, CT-ORG, and MSD-Task03-Liver datasets.

### Generalizability of the FSS-ULivR model

To further validate the generalizability and reliability of the proposed FSS-ULivR model beyond liver segmentation, we conducted comprehensive evaluations on multi-organ segmentation tasks and brain tumor segmentation across multiple datasets. The FSS-ULivR model was evaluated on the CT-ORG dataset using CT images for multi-organ segmentation, including bladder, bones, kidneys, and lungs, as well as on BraTS 2019 and BraTS 2020 datasets, using FLAIR and T1ce modalities, for brain tumor segmentation tasks including whole tumor (WT), tumor core (TC), and enhancing tumor (ET) components.

For multi-organ segmentation on the CT-ORG dataset, FSS-ULivR demonstrated exceptional performance across all anatomical structures. The model achieved outstanding results for bladder segmentation with a Dice coefficient of 93.54±1.47%, IoU of 92.27±1.38%, and specificity of 99.97±0.12%. For bone segmentation, FSS-ULivR obtained a Dice coefficient of 85.93±2.31%, IoU of 78.52±2.89%, and specificity of 99.63±0.19%. Kidney segmentation yielded excellent results with a Dice coefficient of 94.26±1.52%, IoU of 92.51±1.74%, and specificity of 99.76±0.15%. Finally, for lung segmentation, the model achieved a Dice coefficient of 93.15±1.68%, IoU of 91.54±2.12%, and specificity of 99.88±0.11%. Figure 7 illustrates the multi-organ segmentation results on the CT-ORG dataset.

In brain tumor segmentation tasks, FSS-ULivR demonstrated consistent performance across both BraTS datasets. On the BraTS 2019 dataset, the model achieved a Dice coefficient of 93.18±1.74% for whole tumor segmentation, 90.20±2.13% for tumor core, and 88.54±2.47% for enhancing tumor. The corresponding IoU values were 87.26±1.91%, 82.20±2.58%, and 79.46±2.73%, with specificity values of 95.74±1.26%, 96.26±1.41%, and 93.13±1.76%, respectively. On the BraTS 2020 dataset, FSS-ULivR maintained robust performance with Dice coefficients of 92.50±1.89% for whole tumor, 87.46±2.35% for tumor core, and 88.13±2.21% for enhancing tumor, with IoU values of 86.09±2.14%, 77.80±2.97%, and 78.81±2.64%, and specificity values of 95.13±1.38%, 92.61±1.73%, and 93.42±1.74%, respectively.

**Fig. 8:**
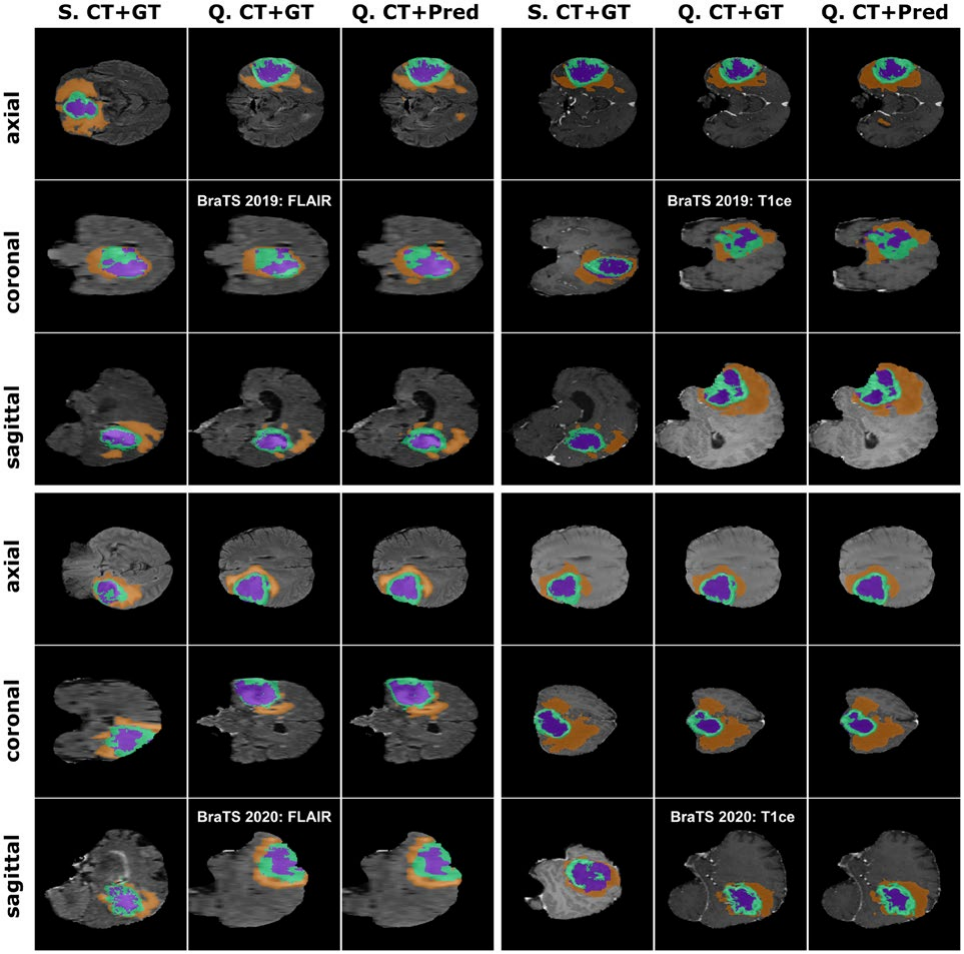
Brain tumor segmentation results of FSS-ULivR model on BraTS 2019 and BraTS 2020 datasets, showcasing the accurate segmentation of whole tumor (WT), tumor core (TC), and enhancing tumor (ET) components. Each row displays different anatomical views (axial, coronal, and sagittal) with columns representing S (Support image), Q (Query image), GT (Ground Truth segmentation), and Pred (Predicted segmentation)

The results consistently demonstrate the superior performance, versatility, and generalizability of FSS-ULivR across diverse anatomical structures and pathological conditions. Figure 8 showcases the brain tumor segmentation performance on both BraTS 2019 and BraTS 2020 datasets, further confirming the model’s exceptional adaptability and clinical applicability. Table 7 presents a comprehensive comparison of FSS-ULivR against state-of-the-art methods, including U-Net (Ronneberger et al. 2015), U-Net++ (Zhou et al. 2018), TransUNet (Chen et al. 2024), and Swin-Unet (Cao et al. 2022) across all evaluated organs and tumor components.

**Table 7:** Multi-organ and multi-dataset generalizability evaluation of FSS-ULivR compared to state-of-the-art methods on CT-ORG, BraTS 2019, and BraTS 2020 datasets using Dice coefficient, Intersection over Union (IoU), and Specificity metrics

Dataset	Labels	Metrics (%)	U-Net (Ronneberger et al. 2015)	U-Net++ (Zhou et al. 2018)	TransUNet (Chen et al. 2024)	Swin-Unet (Cao et al. 2022)	FSS-ULivR
CT-ORG	Bladder	Dice	89.72±2.13	91.05±1.89	92.41±1.76	92.87±1.54	**93.54±1.47**
		IoU	87.38±2.47	89.12±2.31	90.76±1.93	91.25±1.82	**92.27±1.38**
		Specificity	99.81±0.18	99.89±0.14	99.92±0.13	99.94±0.11	**99.97±0.12**
	Bones	Dice	79.67±3.24	81.43±2.89	83.15±2.56	**85.97±2.18**	85.93±2.31
		IoU	72.34±3.87	74.91±3.42	76.58±3.19	77.23±2.94	**78.52±2.89**
		Specificity	99.47±0.26	99.52±0.23	99.58±0.21	99.61±0.18	**99.63±0.19**
	Kidneys	Dice	90.83±2.17	92.19±1.94	93.45±1.73	93.89±1.61	**94.26±1.52**
		IoU	88.47±2.53	89.91±2.28	91.12±2.14	91.68±1.97	**92.51±1.74**
		Specificity	99.64±0.21	99.69±0.18	99.73±0.16	99.75±0.14	**99.76±0.15**
	Lungs	Dice	89.45±2.84	90.78±2.41	91.97±2.13	92.64±1.87	**93.15±1.68**
		IoU	86.92±3.16	88.34±2.89	89.73±2.47	90.41±2.25	**91.54±2.12**
		Specificity	99.72±0.19	99.78±0.16	99.83±0.14	99.86±0.12	**99.88±0.11**
BraTS 2019	Whole Tumor	Dice	88.92±2.47	90.15±2.18	91.67±1.93	92.43±1.76	**93.18±1.74**
		IoU	82.65±2.89	84.37±2.64	85.91±2.43	86.58±2.19	**87.26±1.91**
		Specificity	93.82±1.97	94.56±1.73	95.21±1.54	95.67±1.38	**95.74±1.26**
	Tumor Core	Dice	85.73±2.84	87.19±2.56	88.64±2.31	89.42±2.18	**90.20±2.13**
		IoU	77.39±3.21	79.15±2.97	**82.87±2.76**	81.54±2.63	82.20±2.58
		Specificity	94.18±2.13	94.89±1.94	95.43±1.76	95.91±1.58	**96.26±1.41**
	Enhancing Tumor	Dice	83.47±3.12	85.21±2.87	86.93±2.64	87.72±2.43	**88.54±2.47**
		IoU	74.25±3.58	76.13±3.31	77.84±3.17	78.47±2.94	**79.46±2.73**
		Specificity	91.67±2.34	92.41±2.18	92.98±1.97	**93.29±1.84**	93.13±1.76
BraTS 2020	Whole Tumor	Dice	87.54±2.73	89.12±2.43	90.78±2.16	91.63±1.94	**92.50±1.89**
		IoU	81.29±3.14	83.47±2.87	84.92±2.64	85.73±2.41	**86.09±2.14**
		Specificity	92.84±2.16	93.67±1.93	94.38±1.72	94.89±1.54	**95.13±1.38**
	Tumor Core	Dice	82.19±3.47	84.05±3.12	85.73±2.89	86.58±2.67	**87.46±2.35**
		IoU	72.87±3.94	74.92±3.63	76.41±3.38	77.13±3.21	**77.80±2.97**
		Specificity	90.43±2.56	91.28±2.34	91.97±2.18	92.47±1.94	**92.61±1.73**
	Enhancing Tumor	Dice	82.67±3.28	84.39±2.94	86.15±2.73	87.21±2.56	**88.13±2.21**
		IoU	73.52±3.67	75.84±3.41	77.36±3.19	78.15±2.89	**78.81±2.64**
		Specificity	91.38±2.47	**94**.**15**±**2**.**23**	92.86±2.14	92.56±1.87	93.42±1.74

Bold text represents the highest results

### Ablation study

This section provides insights into ablation experiments (see Sect. 4.3 for the used computational resources) focused on key components of our FSS-ULivR model.

*Loss weight coefficient tuning.* To identify the optimal combination of weighting coefficients $(\alpha, \beta, \gamma, \delta )$ for our composite loss function (see Sect. 3.5), we initially conducted a focused experiment study on the LiTS dataset with 50 epochs (see Table 8).

The coefficients were systematically varied such that their sum remained equal to 1.0, and each configuration was evaluated based on the training and validation Dice scores. The best-performing configuration, which was later adopted in the final model, achieved a (total) training and validation Dice of 0.889 and 0.838, respectively.

**Table 8:** Loss weight coefficient tuning on the LiTS dataset. The best parameters (in 50 epochs) are bolded

Exp.	$\alpha$	$\beta$	$\gamma$	$\delta$	Dice Score	
					Train	Val
0	0.10	0.10	0.10	0.70	0.742	0.705
1	0.15	0.15	0.20	0.50	0.791	0.753
2	0.20	0.20	0.30	0.30	0.824	0.782
3	0.25	0.25	0.30	0.20	0.855	0.809
**4**	**0**.**30**	**0**.**25**	**0**.**30**	**0**.**15**	**0**.**889**	**0**.**838**
5	0.35	0.25	0.25	0.15	0.851	0.804
6	0.40	0.20	0.25	0.15	0.828	0.785

**Fig. 9:**
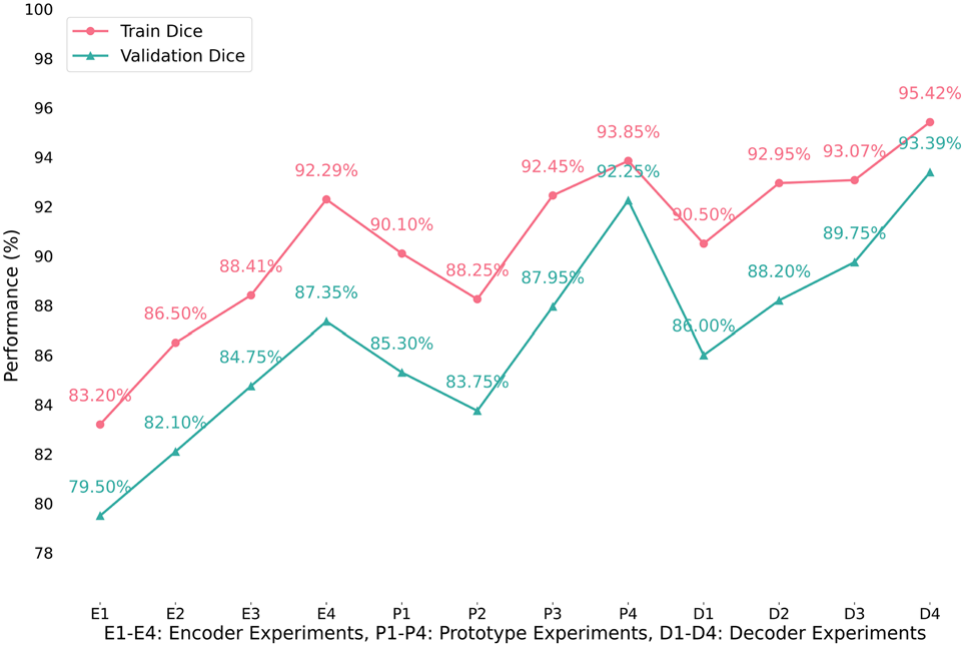
Ablation study results showing training and validation Dice performance across encoder experiments (E1-E4), prototype experiments (P1-P4), and decoder experiments (D1-D4)

*Architectural design.* We conducted an extensive ablation study on the LiTS dataset to systematically assess the impact of each component. The architecture was broken down into three key modules: Encoder, Prototype, and Decoder. For each module, we tested multiple configurations by varying architectural choices and hyperparameters, while keeping the training setup consistent across experiments (100 epochs, Dice-based evaluation). Figure 9 illustrates the performance comparison between training and validation Dice scores across all experimental configurations.

**Table 9:** Summary of architecture ablation experiments on the LiTS dataset (100 epochs). The total inference time (Inf. (m)) is calculated as the multiplication of total epochs (Epc) with each epoch time (T); and Memory includes trainable (MB) and non-trainable (KB) parameters

Com.	Exp.	Settings	LR	Batch	Dim	Epc$\times$T	Dice (%)		Inf. (m)	Memory	Remarks
							Train	Val			
Encoder	E1	ResNet (no SE, baseline)	0.01	4	256$\times$256	100 $\times$ 270 s	83.20	79.50	450	85.5 MB / 66.4 KB	Weakest baseline, low capacity
	E2	ResNet + SE (64–256)	0.005	8	512$\times$512	100 $\times$ 171 s	86.50	82.10	285	93.8 MB / 38.9 KB	Moderate gain, stable setup
	E3	ResNet + SE (Dep.$\uparrow$, 6B)	0.002	16	512$\times$512	100 $\times$ 310 s	88.41	84.75	517	115.9 MB / 59.3 KB	Depth $\uparrow$, but diminishing returns
	E4	ResNet + SE (64–512)	0.001	8	256$\times$256	100 $\times$ 100 s	**92**.**29**	**87**.**35**	167	97.5 MB / 71.2 KB	Best Val Dice, good trade-off
Decoder	D1	Upsample + basic skip	0.01	8	256$\times$256	100 $\times$ 140 s	90.50	86.00	233	102.0 MB / 31.7 KB	Std. baseline
	D2	+ SE-enhanced res blocks	0.005	4	256$\times$256	100 $\times$ 275 s	92.95	88.20	458	110.4 MB / 76.9 KB	Val $\uparrow$, cost $\uparrow$ with SE
	D3	+ Attention Gate (skip-attn fusion)	0.002	16	512$\times$512	100 $\times$ 120 s	93.07	89.75	200	95.0 MB / 88.1 KB	Fastest inf., high Dice
	D4	+ Residual Refinement (full decoder)	0.001	8	256$\times$256	100 $\times$ 230 s	**95**.**42**	**93**.**39**	383	93.1 MB / 61.4 KB	Best Dice, but heaviest overall
Prototype	P1	SE block in support stream	0.01	8	256$\times$256	100 $\times$ 280 s	90.10	85.30	467	102.5 MB / 43.7 KB	Val $\uparrow$, but high latency cost
	P2	Basic (global avg. mask)	0.005	16	256$\times$256	100 $\times$ 150 s	88.25	83.75	250	113.6 MB / 76.5 KB	Best trade-off: speed vs. Dice
	P3	+ SE + Transformer (1-H, 64D)	0.002	8	512$\times$512	100 $\times$ 195 s	92.45	87.95	325	106.0 MB / 38.4 KB	Good jump, cost $\uparrow$
	P4	+ SE + Transformer + Channel Attention	0.001	8	256$\times$256	100 $\times$ 110 s	**93**.**85**	**92**.**25**	183	98.5 MB / 57.2 KB	Most accurate, efficient inf

Bold text represents the highest results

For the encoder, we began with a baseline ResNet without SE blocks, which had the lowest memory footprint but also the weakest performance. We noticed that adding SE blocks led to improved accuracy and a moderate increase in memory (from 85.5 MB to 93.8 MB). Increasing the model depth also increased Dice scores, but also significantly raised the memory usage to 115.9 MB. The wider channel range offered the best trade-off and produced the highest validation Dice with only a slight memory increase compared to the SE baseline. For the decoder, the basic upsampling setup used moderate memory. Enhancing it with SE-enhanced residual blocks increased memory to 110.4 MB, while attention gating achieved better accuracy with slightly less memory usage (95.0 MB). Interestingly, the final addition of a residual refinement stage boosted Dice scores to their peak while reducing memory to 93.1 MB. In the prototype module, the basic global averaging setup consumed relatively high memory (113.6 MB), but adding SE and a lightweight Transformer slightly reduced memory to 106.0 MB. The full configuration, with SE, Transformer, and channel attention, not only achieved the highest Dice but also reduced memory to 98.5 MB, which makes it both the most accurate and memory-efficient. The detailed ablation experiment results are summarized in Table 9.

*Hyperparameters tuning*. For hyperparameter tuning, the optimal configuration is with a learning rate of 0.001, a batch size of 8, the Adam optimizer, a dropout of 0.3, and image dimensions of 256$\times$256. After this experiment, the final model was experimented with 200 epochs and delivered 98.94±0.24% Dice coefficient, 97.44±1.09% IoU, and 93.78±3.72% specificity on the best selected configurations.

## Discussion

Liver segmentation in few-shot scenarios poses unique challenges, including robust feature extraction from minimal data, effective integration of anatomical context, and precise delineation of liver boundaries. While recent studies have proposed hybrid architectures such as ResUNet (Rahman et al. 2022), H-DenseUNet (Li et al. 2018), and Un-Net (Tran et al. 2020) to enhance spatial-contextual representation, and and attention-based decoders like mU-Net (Seo et al. 2019) and AHCNet (Jiang et al. 2019) to recover fine details, several approaches still require abundant annotations or struggle with generalization. To address these challenges, we propose FSS-ULivR, a novel few-shot segmentation framework that effectively combines a ResNet-based encoder enhanced with SE modules, an Enhanced Prototype Module utilizing transformer-based self-attention and channel refinement, and a decoder with improved attention gating and residual refinement strategies. Extensive evaluations on the LiTS dataset and several external benchmarks, including 3DIRCADB01, CRLM, CT-ORG, and MSD-Task03-Liver, underscore the robustness and generalizability of our approach. The results, as detailed in Tables 3, 5 and 6 and illustrated in Figs. 5 and 6, demonstrate that our FSS-ULivR framework outperforms comparative methods in both qualitative and quantitative evaluations, highlighting its potential to facilitate more precise diagnosis and treatment planning in clinical settings.

### Discussion on encoder

Our encoder employs a ResNet backbone integrated with SE modules to dynamically recalibrate channel-wise features. This is particularly important in few-shot scenarios, where limited annotated data makes it difficult to learn robust representations. In liver CT scans, different anatomical structures often share similar intensity values, making it challenging for standard convolutional layers to differentiate liver tissue from surrounding organs. SE modules mitigate this by selectively emphasizing informative channels and suppressing less relevant ones, thereby adapting to the specific characteristics of each input image. This design not only improves the discriminative power of extracted features but also helps reduce information loss from aggressive downsampling, a common limitation in standard convolutional encoders (Rahman et al. 2022; Tran et al. 2020). The SE modules further help preserve crucial spatial hierarchies and emphasize clinically relevant details, thereby improving the overall segmentation quality. This channel-wise recalibration is particularly beneficial for liver segmentation because it enables the model to automatically focus on texture patterns and intensity variations that are most relevant for delineating liver boundaries, even when these patterns are subtle or occur infrequently in limited data scenarios. As shown in Fig. 3A and B, our encoder significantly enhances feature representation for both support and query images when compared to baseline methods. This strong performance is particularly useful considering the variations in liver CT scans and the limited amount of annotated data available in few-shot scenarios.

### Discussion on prototype module

As shown in Fig. 2, our prototype module integrates a transformer block that helps capture long-range dependencies and global context, which are essential for generating robust support prototypes. The motivation for incorporating transformer blocks in the prototype module arises from a fundamental limitation of traditional prototype-based few-shot segmentation methods, which rely on local feature averaging and often fail to capture the global anatomical context essential for accurate liver segmentation. Liver structures display complex morphologies and may be partially visible in 2D slices, requiring the model to understand spatial relationships across distant regions. The self-attention mechanism in transformer blocks enables the model to establish these long-range dependencies, allowing it to generate more consistent and anatomically plausible prototypes even when only limited support examples are available. The addition of channel attention further refines the feature maps, enabling the model to focus on informative regions while filtering out background noise. Channel attention is motivated by the observation that different feature channels encode different types of anatomical information, and in few-shot settings, it becomes crucial to identify which channels carry the most discriminative information for the target anatomy. By learning channel-wise importance weights, the model can adaptively emphasize features that are most relevant for liver segmentation while suppressing those that might be influenced by variations in imaging protocols or patient anatomy. Although influenced by recent transformer-based and attention-guided segmentation methods (Zhang et al. 2022; Jiang et al. 2019), our model provides a more effective mechanism for fusing refined support and query features, bridging the semantic gap between support and query images even under severe data scarcity (Alsaleh et al. 2024; Chen et al. 2024).

### Discussion on decoder

Our decoder is designed to restore spatial details lost during downsampling by using advanced attention gates and residual refinement strategies to merge multi-scale contextual information from encoder skip connections. The need for improved attention gates arises because conventional skip connections in U-Net architectures often carry irrelevant features from earlier encoder layers, which is particularly problematic in few-shot scenarios where the model has limited exposure to diverse anatomical variations. The attention gates work by selectively combining features from different scales, ensuring that only the most relevant spatial information is passed on to the final segmentation. This selective propagation is particularly important for liver segmentation, as the organ’s boundaries frequently overlap with adjacent structures, requiring the model to distinguish important anatomical details from distracting or irrelevant information. This approach follows recent advances in attention-based multi-scale feature fusion (Jiang et al. 2019; Liu et al. 2024), providing improved anatomical detail recovery and better results than traditional methods through the additional incorporation of residual connections. Residual refinement techniques are introduced to help retain detailed features while ensuring overall consistency in the segmentation. In few-shot liver segmentation tasks, accurately capturing the boundaries is often challenging, particularly where the liver interfaces with surrounding tissues. By incorporating residual connections within the decoder, the model can progressively improve its predictions, using detailed spatial information from various encoder layers to correct and enhance unrefined outputs. Ablation studies in Table 9 indicate that these decoder improvements effectively capture both global context and local fine details, which are essential for obtaining precise segmentation results in clinical applications.

### Discussion on FSS-ULivR

The FSS-ULivR framework combines a ResNet-based encoder with SE modules, a transformer-enhanced prototype module, and a decoder with refined attention gating to address the challenges of few-shot liver segmentation. The overall architectural design is motivated by the need to overcome critical difficulties in few-shot medical image segmentation, including effective feature extraction from limited data, generating prototypes that preserve anatomical consistency, and recovering spatial details to ensure clinical accuracy. Each component is carefully designed to address a specific aspect of these difficulties while working together to enhance the model’s overall performance. The encoder robustly extracts discriminative features while preserving spatial hierarchies via SE modules (Rahman et al. 2022; Tran et al. 2020), motivated by the need for adaptive feature selection in data-scarce scenarios, the prototype module utilizes transformer-based self-attention and channel refinement to bridge semantic gaps between support and query images, even in limited data scenarios (Zhang et al. 2022; Jiang et al. 2019), motivated by the requirement for global context understanding in anatomical segmentation, and the decoder restores precise anatomical details through multi-scale attention fusion and residual refinements, surpassing traditional methods (Liu et al. 2024), motivated by the clinical need for accurate boundary delineation in diagnostic applications. These integrations significantly improve segmentation quality, demonstrating superior performance across diverse benchmarks, including 3DIRCADB01, CRLM, CT-ORG, and MSD-Task03-Liver, BraTS 2019, BraTS 2020, and highlighting the potential for enhancing clinical diagnostic processes.

The comprehensive integration of encoder with SE-enhanced residual blocks, Enhanced Prototype Module, and attention-guided decoder in FSS-ULivR represents a substantial advancement over existing methods by addressing fundamental limitations in few-shot medical image segmentation. The motivation for SE-enhanced residual blocks arises from the need to adaptively recalibrate feature channels while preserving gradient flow in few-shot scenarios, where standard residual connections fail to emphasize discriminative anatomical features necessary for liver boundary detection. Unlike traditional approaches relying on simple prototype averaging, our Enhanced Prototype Module is motivated by the observation that liver segmentation requires both local texture understanding and global anatomical context, a dual requirement that conventional prototype methods cannot satisfy. The integration of transformer-based self-attention within prototype computation addresses the need to establish long-range spatial dependencies across liver regions that may be spatially disconnected in 2D slices, while channel attention identifies which feature channels carry the most relevant anatomical information when training data is severely limited. Our decoder’s attention gates are motivated by the clinical requirement for precise boundary delineation, where traditional skip connections often propagate irrelevant background features that compromise segmentation accuracy, and their integration with residual refinement provides superior boundary recovery compared to standard U-Net variants by enabling progressive feature enhancement while maintaining spatial detail preservation essential for clinical applications. This substantial improvement is evidenced by consistent performance gains across multiple challenging datasets, demonstrating superior generalizability and addressing the critical gap where existing methods either require extensive data or compromise segmentation precision for clinical applications.

## Conclusion

In this study, we have presented the FSS-ULivR framework to address the challenges related to precise liver segmentation with minimal annotated data. Our model is specifically designed for few-shot segmentation and integrates a ResNet-based encoder with an enhanced prototype-based few-shot learning module. This module employs a transformer block for global feature representation and an SE block for dynamic channel-wise feature refinement. In addition, attention mechanisms in the decoder effectively focus on essential regions, thereby enabling precise segmentation even in challenging scenarios. Through extensive experiments, the FSS-ULivR model achieved an outstanding Dice coefficient of 98.94%, Intersection over Union (IoU) of 97.44% and a specificity of 93.78% on the LiTS dataset, demonstrating its capability to generate precise and reliable segmentation results. To assess the generalizability of our approach, we conducted cross-dataset evaluations on four external datasets: 3DIRCADB01, CRLM, CT-ORG, and MSD-Task03-Liver. The model consistently delivered high Dice coefficients and specificity scores across all datasets, achieving 95.43% Dice, 92.20% IoU, and 93.81% specificity on 3DIRCADB01, 92.98% Dice, 90.16% IoU, and 95.93% specificity on CRLM, 90.72% Dice, 86.47% IoU, and 94.85% specificity on CT-ORG, and 94.05% Dice, 89.32% IoU, and 91.53% specificity on MSD-Task03-Liver. To further validate the model’s generalizability and reliability across different anatomical structures, we extended our evaluation to multi-organ segmentation on the CT-ORG dataset, achieving 93.54% Dice, 92.27% IoU, and 99.97% specificity for bladder, 85.93% Dice, 78.52% IoU, and 99.63% specificity for bones, 94.26% Dice, 92.51% IoU, and 99.76% specificity for kidneys, and 93.15% Dice, 91.54% IoU, and 99.88% specificity for lungs. Additionally, we evaluated the model’s capability on brain tumor segmentation using BraTS 2019 and BraTS 2020 datasets for whole tumor, tumor core, and enhancing tumor regions, achieving average performance of 90.64% Dice, 82.97% IoU, and 95.04% specificity on BraTS 2019, and 89.36% Dice, 80.90% IoU, and 93.72% specificity on BraTS 2020. These results highlight the ability of FSS-ULivR to deliver precise and reliable liver segmentation across varying clinical imaging protocols, without requiring additional training or fine-tuning. This strong performance underscores its promise for integration into real-world clinical workflows. However, the current evaluation is limited to publicly available datasets, which may not fully represent the variability encountered in clinical practice. While the proposed model has been evaluated on both CT and MRI modalities, it remains to be validated on a broader spectrum of multimodal imaging data, such as PET, ultrasound, and histopathology. To address these limitations, future work will involve collecting large-scale real patient data and incorporating diverse imaging modalities to more rigorously assess the model’s robustness and applicability in heterogeneous, real-world clinical settings.

## Data Availability

We utilized seven publicly available datasets in this study, including the Liver Tumor Segmentation Challenge (LiTS) dataset (Bilic et al. 2023), the 3D-IRCADb-01 dataset (Soler et al. 2015), the Colorectal Liver Metastases (CRLM) dataset (Simpson et al. 2024), the Computed Tomography Organs (CT-ORG) dataset (Rister et al. 2019), and the Medical Segmentation Decathlon Challenge Task 3: liver (MSD-Task03-Liver) dataset (Antonelli et al. 2022), as well as the Brain Tumor Segmentation Challenge 2019 (BraTS 2019) dataset (Bakas et al. 2017) and the Brain Tumor Segmentation Challenge 2020 (BraTS 2020) dataset (Menze et al. 2014).

## Footnotes

1 https://academictorrents.com/details/27772adef6f563a1ecc0ae19a528b956e6c803ce

2 https://www.ircad.fr/research/data-sets/liver-segmentation-3d-ircadb-01/

3 https://www.cancerimagingarchive.net/collection/colorectal-liver-metastases/

4 https://www.cancerimagingarchive.net/collection/ct-org/

5 https://drive.google.com/file/d/1jyVGUGyxKBXV6_9ivuZapQS8eUJXCIpu/view?usp=sharing

6 https://www.med.upenn.edu/cbica/brats2019/data.html

7 https://www.med.upenn.edu/cbica/brats2020/data.html
